# Predictive value of the combined cholesterol, high-density lipoprotein, glucose and frailty indices for cardiometabolic multimorbidity incidence: evidence from a national prospective cohort study

**DOI:** 10.1186/s12933-026-03213-0

**Published:** 2026-05-17

**Authors:** Huanqiong Fan, Guosong Jiang, Jun Cheng, Hengfa Chen

**Affiliations:** 1 Department of Pharmacy, Foshan University Affiliated Third Hospital, Foshan, Guangdong China; 2 Department of Pulmonary and Critical Care Medicine, Northeast Yunnan Central Hospital, Northeast Yunnan Hospital of Kunming Medical University, Northeast Yunnan Clinical School of Kunming Medical University, Zhaotong, 657000 Yunnan China; 3 Kunming Medical University, Kunming, 650500 Yunnan China; 4 Department of cardiovascular medicine, Foshan University Affiliated Third Hospital, Foshan, Guangdong China; 5 School of Artificial Intelligence, South China Normal University, Guangzhou, Guangdong China

**Keywords:** Cholesterol, High-density lipoprotein, Glucose, Frailty index, Cardiometabolic multimorbidity, China health, Retirement longitudinal survey

## Abstract

**Background:**

The cholesterol, high-density lipoprotein, and glucose (CHG) is a surrogate of insulin resistance, while frailty reflects cumulative physiological decline, yet their combined utility for cardiometabolic multimorbidity (CMM) is underexplored. We evaluated a combined CHG–frailty index (CHG–FI) for incident heart disease, stroke, diabetes, and CMM.

**Methods:**

We conducted a retrospective cohort study of 6812 Chinese adults aged ≥ 45 years enrolled in the 2011–2020 waves of the China Health and Retirement Longitudinal Study (CHARLS). Participants with baseline lipid and fasting glucose measurements were included and followed prospectively for incident heart disease, stroke, diabetes, and cardiometabolic multimorbidity (≥ 2 conditions). FI was calculated using the cumulative deficit approach, and CHG was incorporated according to established procedures. Multivariable Cox regression estimated associations; CHG-FI interaction was assessed on multiplicative and additive scales. Incremental predictive utility of CHG, FI, TYG‑FI, and CHG‑FI was compared using receiver operating characteristic (ROC) curves, net reclassification improvement (NRI), and decision curve analysis (DCA). Stratified and sensitivity analyses evaluated robustness.

**Results:**

Over a median follow‑up of 9.0 years, we observed 1,304 heart disease events, 554 strokes, 932 diabetes cases, and 467 CMM cases. Each 1-unit increase in CHG-FI was associated with higher risk: heart disease (HR 1.34, 95% CI 1.23–1.45), stroke (HR 1.85, 95% CI 1.65–2.06), diabetes (HR 1.29, 95% CI 1.17–1.42), and CMM (HR 1.79, 95% CI 1.58–2.04). Associations showed dose–response patterns and nonlinearity. The multiplicative interaction between CHG and FI for CMM was 0.54 (95% CI 0.35–0.83), reflecting a “risk saturation” or ceiling effect. Despite this, the combined CHG -FI index offered superior predictive performance for CMM relative to individual components: AUC 0.652 (95% CI 0.627–0.678), with significant improvement in reclassification (NRI 0.329, 95% CI 0.236–0.423) and discrimination (IDI 0.011, 95% CI 0.008–0.015). CHG -FI and TyG -FI showed broadly comparable performance across outcomes.

**Conclusion:**

The combined CHG-frailty index was significantly associated with incident cardiometabolic multimorbidity and its individual components, demonstrating predictive performance comparable to established combined indices. As an accessible tool integrating routine metabolic and physiologic reserve measures, CHG-FI offers a practical alternative approach for risk stratification.

**Graphical abstract:**

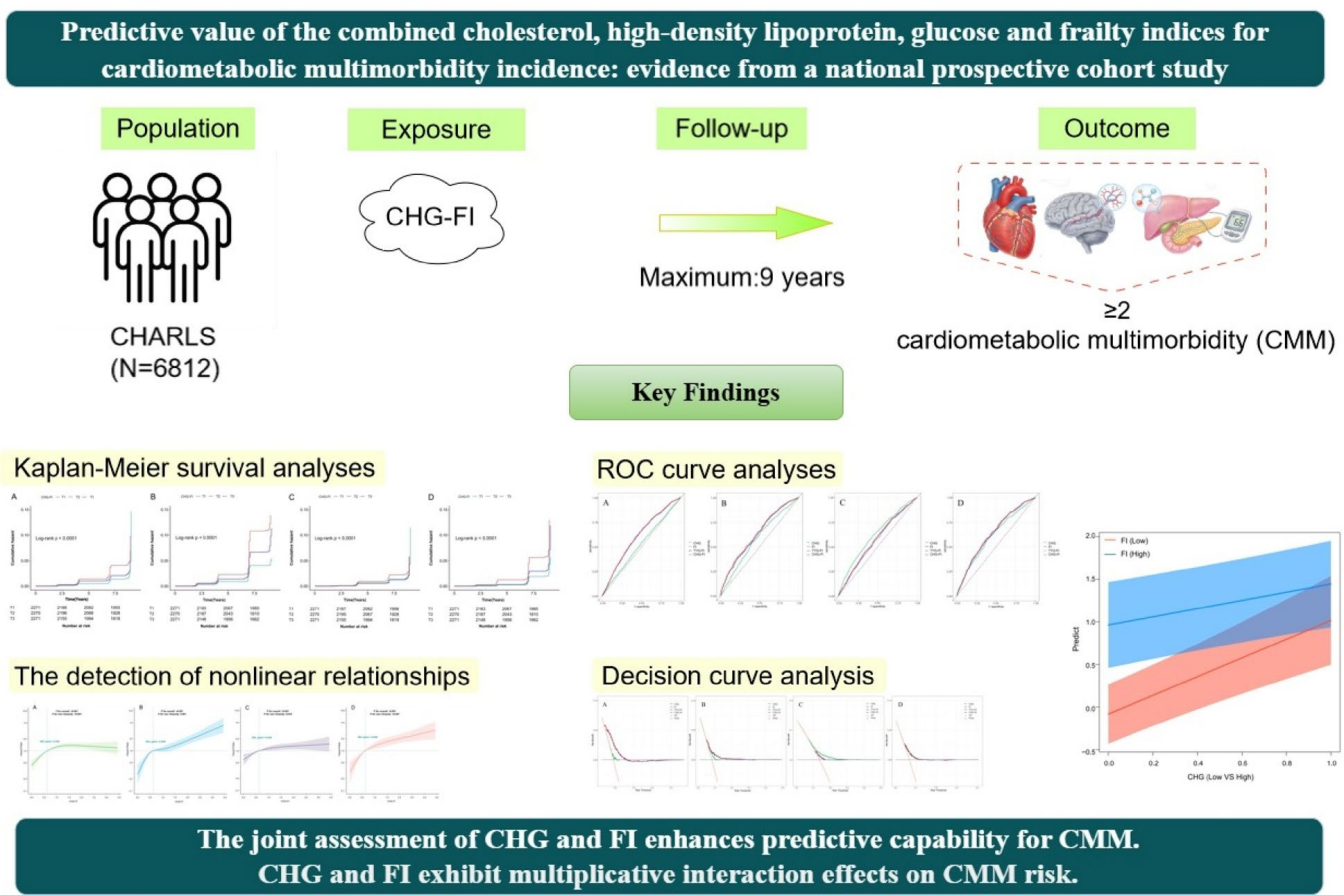

**Supplementary Information:**

The online version contains supplementary material available at 10.1186/s12933-026-03213-0.

## Research insights

**What is currently known about this topic?**

- The TyG index and related biomarkers are established surrogates of insulin resistance. The frailty index (FI) is a validated measure of multi-system physiological vulnerability. Both predict adverse cardiometabolic outcomes. However, the combined utility of a cholesterol, high-density lipoprotein, and glucose index (CHG) and the FI for predicting cardiometabolic multimorbidity (CMM) remains under-explored.

**What is the key research question?**

- Does the CHG‑FI improve prediction of incident heart disease, stroke, diabetes, and CMM? Do CHG and FI interact on multiplicative or additive scales with respect to CMM risk?

**What is new?**

- The CHG-FI was independently associated with higher risks of heart disease, stroke, diabetes, and CMM, demonstrating dose-response, nonlinear relationships and a sub-multiplicative interaction between CHG and frailty index for CMM.

**How might this study influence clinical practice?**

- By combining metabolic and frailty domains and capturing mechanisms distinct from TyG-FI, the CHG-FI identifies vulnerable middle-aged and older adults across a broad range of dyslipidemia profiles, enabling earlier, targeted interventions to prevent incident CMM.

## Introduction

Cardiometabolic diseases, primarily heart disease, stroke, and type-2 diabetes, pose a massive global health burden. When two or more of these conditions coexist, the syndrome is defined as cardiometabolic multimorbidity (CMM) [1, 2]. In China, rapid population aging, adverse lifestyle changes, and shifts in cardiometabolic risk profiles have driven a sustained increase in CMM prevalence [3]. Compared with single diseases, the clustering of cardiometabolic disorders predicts steeper functional decline, higher healthcare utilization, and an exponentially increased risk of mortality [4, 5]. While traditional risk factors partially explain cardiovascular susceptibility, they inadequately capture the complex, multi-system physiological derangements that precipitate the compounding transition from a single disease to CMM [6–7]. Consequently, there is an urgent need for composite biomarkers that integrate broader dimensions of metabolic and biological aging.

The triglyceride-glucose (TyG) index, derived from fasting triglycerides and fasting glucose, is a well-established surrogate for insulin resistance and cardiovascular risk [8]. However, because it is heavily weighted toward triglyceride levels, the TyG index primarily captures adipose-related ectopic fat deposition. This creates a predictive limitation for older adults with normal triglyceride levels, in whom atherosclerotic risk may be driven primarily by cholesterol–glucose metabolic pathways rather than triglyceride-mediated mechanisms [9–10]. To address this, the cholesterol, high-density lipoprotein, and glucose (CHG) index was recently proposed [11–13]. Unlike TyG, CHG mathematically integrates the atherogenic balance (total cholesterol-to-HDL-C ratio) with fasting glucose. Therefore, CHG captures a distinct pathophysiological axis dominated by cholesterol-mediated vascular burden and glucotoxicity, expanding the toolkit for risk assessment.

Furthermore, metabolic dysregulation rarely acts in isolation; it interacts dynamically with the aging process. Frailty, quantified by the Frailty Index (FI), reflects a cumulative decline in physiological reserve and multi-system vulnerability that cannot be inferred from chronological age alone [14, 15]. Biologically, metabolic stress (e.g., glucotoxicity, lipotoxicity) and frailty share converging pathological pathways, such as chronic low-grade inflammation, oxidative stress, and endothelial dysfunction [16–18]. We hypothesize that these overlapping mechanisms render frail individuals disproportionately vulnerable to metabolic insults. Therefore, integrating high metabolic stress with depleted physiological reserve could provide a more holistic evaluation of an individual’s vulnerability [19, 20].

While the combination of TyG and FI has been recently explored for predicting isolated vascular events [21], no prior research has evaluated a combined metabolic-frailty framework specifically for the incidence of CMM. To address this critical gap, the present study utilizes data from a large, nationally representative prospective cohort with the following specific aims: (1) to evaluate the predictive performance of the combined CHG-FI index for incident CMM and its individual components (heart disease, stroke, and diabetes); (2) to formally dissect the interaction pattern between the metabolic and frailty domains to understand their joint effects; (3) to benchmark CHG-FI against the established TyG-FI, demonstrating its phenotypic complementarity for clinical use; and (4) to verify the robustness of CHG-FI across prespecified demographic and clinical subgroups.

## Materials and methods

### Data sources and study population

We used five biennial waves (2011–2020) of the China Health and Retirement Longitudinal Study (CHARLS) [22]. CHARLS targets Chinese adults aged ≥ 45 years. CHARLS was designed to study population ageing. It also monitors changes in health, economic status, and social conditions among middle-aged and older adults. The survey used a four‑stage stratified cluster sampling scheme. Selection was probability‑proportional‑to‑size (PPS). Participants were enrolled from 450 community units. These units were within 150 county-level jurisdictions across 28 provinces. Biennially, trained teams performed standardize physical examinations. They collected blood specimens for biomarker measurement.

This work is a retrospective cohort study using secondary (post-hoc) analysis of prospectively collected data from the China Health and Retirement Longitudinal Study (CHARLS). While data collection followed a prospective cohort design, the specific hypothesis, CHG-FI index construction, and analytical plan were formulated after data collection was completed. This retrospective approach carries inherent risks of over-fitting and data-driven specification, which we addressed through per-specified analytical protocols and sensitivity analyses.

This study employed the DE-identified, publicly available CHARLS datasets. Ethical approval for the original CHARLS data collection was obtained from the Biomedical Ethics Review Committee of Peking University (IRB00001052-11015). For the secondary analysis of individual-level data, the study protocol and data management plan were additionally reviewed and approved by the Ethics Committee of South China Normal University (approval number: SCNU-SAI-2026-008), ensuring that the analytical procedures, data handling, and data governance adhered to relevant local ethical and legal requirements. All analyses were performed in accordance with the ethical standards and protocols established by the CHARLS study.

**Fig. 1:**
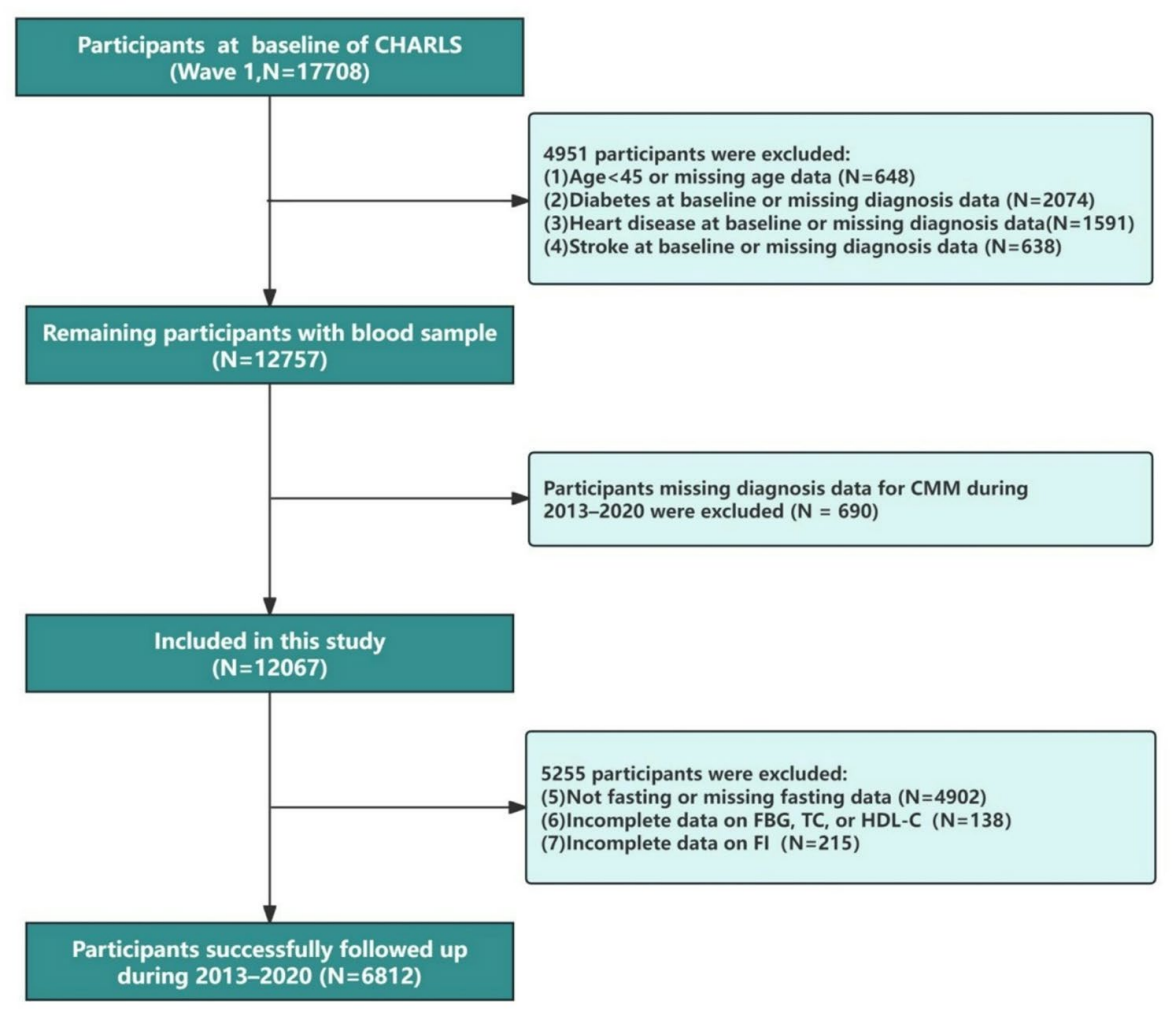
Flow-diagram illustrating the participant selection process. CMM, Cardiometabolic multimorbidity; FBG, Fasting blood glucose; TC, Total cholesterol; HDL-C, High-density lipoprotein cholesterol; FI, Frailty index

Figure 1 shows the selection process. Applying inclusion and exclusion criteria yielded a final sample of 6,812 analyzable individuals from the 2011–2012 cohort. Follow-up assessments occurred from 2013 to 2020.

## Assessment of CMM

The primary outcome was incident cardiometabolic multimorbidity (CMM). CMM was defined as the co-occurrence of two or more cardiometabolic disorders: heart disease, stroke, or diabetes [23].

Heart disease and stroke were ascertained primarily by participant self-report at baseline and follow-up interviews. Standardized interview questions asked whether a doctor had ever diagnosed the respondent with coronary heart disease, angina, congestive heart failure, or any other heart problem, and whether a doctor had ever diagnosed a stroke. Documented use of cardiovascular medications (including anti-platelet agents, anticoagulants, nitrates, and heart failure medications) was used as supplementary information to support self-reported diagnoses but was not used as an independent criterion for identifying cases. Diabetes was classified if any of the following criteria were met: (1) self-reported physician diagnosis of diabetes or hyperglycemia; (2) current use of glucose-lowering medication; (3) fasting blood glucose ≥ 126 mg/dL; or (4) hemoglobin A1c (HbA1c) ≥ 6.5%. Event time was the interval between the previous interview and the interview at which a new CMM diagnosis was first reported. Participants who did not report CMM were censored at their last survey date. Followup time was calculated from baseline to that final interview [22]. This CMM definition is consistent with prior CHARLS studies [24].

## CHG-FI definition

Frailty was measured using the 32-item Frailty Index (32‑FI) (Supplementary Table S1). It excluded heart disease, stroke, and diabetes, as these conditions are diagnostic criteria for CMM and thus were excluded. It covered comorbidity, disability, depressive symptoms, somatic function, and cognitive performance. Items were coded as 0 (deficit absent) or 1 (deficit present). The cognitive item was scored on a continuous 0–1 scale. Higher values denote worse cognition. We calculated each individual’s FI as the sum of deficits divided by 32 [25].

We computed the CHG index as CHG = ln [TC (mg/dL) × FBG (mg/dL) /2 × HDL (mg/dL)] [11]. The combined metric was constructed multiplicative. It was defined as $\:\mathrm{C}\mathrm{H}\mathrm{G}-\mathrm{F}\mathrm{I}=\mathrm{C}\mathrm{H}\mathrm{G}\times\:\mathrm{F}\mathrm{I}$. This product term follows prior epidemiological practice. It captures potential interaction between metabolic dysregulation and functional deficits [26].

## Covariates

Covariates included age, sex, type of residence, education, marital status, smoking, alcohol use, cancer, chronic kidney disease (CKD), low-density lipoprotein cholesterol (LDL‑C), hemoglobin, C‑reactive protein (CRP), and creatinine. The residence was classified as urban or rural. Education was grouped as no formal schooling, primary school, middle school, or high school or above. Marital status was coded as married versus other. Smoking and alcohol use were recorded as binary (yes/no). Cancer and CKD were ascertained by self-reported physician diagnosis (yes/no).

The triglyceride–glucose (TyG) index was calculated as $TyG = \ln \left[ {TG(mg/dL} \right) \times FBG(mg/dL)/2]$.We defined TyG-FI as Ty${\mathrm{G}} - {\mathrm{FI}} = TyG \times FI.$ This follows prior studies [27].

## Handling of missing data and data preprocessing

Supplementary Table S2 shows the extent of missing data. Before model fitting, we assessed multicollinearity using the generalized variance inflation factor (GVIF). For variables with multiple degrees of freedom, we examined GVIF^1/2Df^. We regarded values ≥ 2 as indicative of problematic collinearity (see Supplementary Table S3). To reduce bias from missingness and to preserve sample size, we performed multiple imputation. We formally assessed the missing data mechanism prior to multiple imputation by applying Little’s test for missing completely at random (MCAR). The test results (χ²= 47.41, df = 47, *p* = 0.456) provided no evidence to reject the null hypothesis of MCAR, indicating that the observed pattern of missingness was compatible with MCAR. Based on this assessment, we performed multiple imputation and generated five completed datasets. The imputation model included key covariates and outcome variables. We combined estimates from the imputed datasets using Rubin’s rules.

### Statistical analyses

We stratified participants into tertiles of baseline CHG‑FI. We assessed normality of continuous variables using the Shapiro-Wilk test. Variables approximating normality were reported as mean ± SD. They were compared by one-way ANOVA. Skewed variables were reported as median (IQR). They were compared by the Kruskal–Wallis test. Categorical variables were presented as counts (percentages). They were compared by χ² tests. All tests were two-sided. *P* < 0.05 was considered significant.

We evaluated associations of CHG‑FI with four outcomes. These were incident heart disease, stroke, diabetes, and CMM. We used Cox proportional hazards models to estimate hazard ratios (HRs) and 95% confidence intervals (CIs). We analyzed CHG‑FI as a continuous variable and as tertiles. We plotted Kaplan–Meier curves for CMM incidence across CHG‑FI tertiles. We fitted a crude model first. We then fitted two adjusted models. Model 1 adjusted for age, sex, marital status, education, residence, smoking, and alcohol use. Model 2 additionally adjusted for cancer, CKD, LDL‑C, C‑reactive protein (CRP), hemoglobin, and creatinine. We tested the proportional hazards assumption using Shenfield residuals (Supplementary Table S4).

We assessed dose–response relations using restricted cubic spline analysis. We placed four knots at the 5th, 35th, 65th, and 95th percentiles. Models adjusted for covariates in Model 2. We examined interaction between CHG and FI on incident CMM. We assessed both multiplicative and additive scales. We tested multiplicative interaction by including a product term in Cox models. This estimated the interaction hazard ratio (HR) with 95% CIs. We quantified additive interaction using the relative excess risk due to interaction (RERI), attributable proportion (AP), and the synergy index (SI). We calculated 95% CIs to assess departure from additivity.

We compared predictive performance of CHG, FI, TYG-FI, and CHG-FI. We used ROC curves, time-dependent receiver operating characteristic, net reclassification improvement (NRI), integrated discrimination improvement (IDI), and decision curve analysis (DCA). We conducted prespecified subgroup analyses by age (< 65 vs. ≥ 65 years), sex, alcohol use, smoking status, CKD, lipid-lowering therapy, and anti-hypertensive therapy. These variables were selected based on: (1) established modifiers of cardiometabolic risk (age, sex); (2) lifestyle factors affecting metabolic and frailty trajectories (alcohol, smoking); (3) organ dysfunction affecting drug metabolism and disease progression (CKD); and (4) treatment status as indicators of disease severity and healthcare access (lipid-lowering and anti-hypertensive therapy). We defined lipid-lowering therapy as current use of statins, fibrates, or other lipid-modifying agents; anti-hypertensive therapy as current use of any anti-hypertensive medication class. Participants without medication were classified as “No”. These definitions capture treated but potentially uncontrolled disease, representing intermediate risk states between untreated and optimally controlled conditions. We estimated subgroup associations for each outcome using multi-variable Cox models adjusted for baseline covariates. We tested interaction between continuous CHG‑FI and subgroup variables using the Wald test in fully adjusted models. Pinteraction < 0.05 indicated statistical significance.

We performed a series of sensitivity analyses to assess the robustness of our findings: (1) restricting analyses to complete cases (no multiple imputation); (2) excluding participants with baseline hypertension, cancer, or dyslipidemia ; (3) excluding participants using anti-hypertensive, glucose-lowering, or lipid-lowering medications; (4) excluding conditions with metabolic abnormalities from the FI by removing hypertension from the 32-item Frailty Index and recalculating the CHG-FI; (5) excluding self-reported histories of malignant tumors from the 32-item FI and recalculating the FI to avoid double-counting cancer as both an FI component and a covariate; (6) adding body mass index (BMI) to the fully adjusted model to control for its potential confounding; (7) applying an alternative definition of CKD in the fully adjusted model to determine whether results depend on CKD classification. In this alternative definition, CKD was defined as self-report or estimated glomerular filtration rate (eGFR) < 60 mL/min/1.73 m².(8) excluding participants who developed heart disease, stroke, diabetes, or CMM within the first two years of follow‑up.(9) conducting competing-risk analyses with the Fine-Gray sub-distribution hazard model, treating non-CMM deaths as competing events; and (10) calculating E-values to quantify the potential impact of unmeasured confounding on the associations between CHG-FI and the four major outcomes. Results for sensitivity checks are reported in Supplementary Table S5–S14.

All analyses were performed in R version 4.2.2 (R Foundation) and Free Statistics v2.1.1 (Beijing Free Clinical Medical Technology Co., Ltd.).

## Results

### Baseline characteristics of the study population

Table 1 reports baseline characteristics of 6,812 participants. Higher CHG-FI tertiles were associated with progressively worse demographic, metabolic, and clinical profiles. Individuals in the highest tertile (T3) were older than those in T1 (61.95 ± 9.65 vs. 56.24 ± 8.50 years; *p* < 0.001). T3 had a higher proportion of women (62.09% vs. 44.56%; *p* < 0.001). T3 also had a higher percentage with no formal education (63.10% vs. 33.42%; *p* < 0.001). Metabolic indicators showed a consistent gradient across CHG-FI groups. Compared with T1, T3 had higher FBG, TC, TG, LDL-C, CRP, TyG, FI, and CHG. Hemoglobin and creatinine were lower in T3. All differences were statistically significant (*p* < 0.001). No significant differences were found for cancer prevalence or HDL-C.

**Table 1:** Baseline characteristics stratified by CHG-FI tertiles

Variables	Total	T1(< 0.462)	T2 (0.462–0.872)	T3 (> 0.872)	*P*-value
No.	6812	2271	2270	2271	
Age (years)	58.82 ± 9.36	56.24 ± 8.50	58.27 ± 9.00	61.95 ± 9.65	< 0.001
Sex, *n* (%)					< 0.001
Female	3601 (52.86)	1012 (44.56)	1179 (51.94)	1410 (62.09)	
Male	3211 (47.14)	1259 (55.44)	1091 (48.06)	861 (37.91)	
Education, *n* (%)					< 0.001
No formal education	3245 (47.64)	759 (33.42)	1053 (46.39)	1433 (63.10)	
Primary school	1451 (21.30)	520 (22.90)	481 (21.19)	450 (19.82)	
Middle school	1369 (20.10)	605 (26.64)	479 (21.10)	285 (12.55)	
High school or above	747 (10.97)	387 (17.04)	257 (11.32)	103 (4.54)	
Marry, *n* (%)					< 0.001
No	779 (11.44)	155 (6.83)	243 (10.70)	381 (16.78)	
Yes	6033 (88.56)	2116 (93.17)	2027 (89.30)	1890 (83.22)	
Rural, *n* (%)					< 0.001
No	2384 (35.00)	945 (41.61)	852 (37.53)	587 (25.85)	
Yes	4428 (65.00)	1326 (58.39)	1418 (62.47)	1684 (74.15)	
Drinking status, *n* (%)					< 0.001
NO	4119 (60.47)	1317 (57.99)	1354 (59.65)	1448 (63.76)	
Yes	2693 (39.53)	954 (42.01)	916 (40.35)	823 (36.24)	
Smoking status, *n* (%)					< 0.001
NO	4154 (60.98)	1287 (56.67)	1377 (60.66)	1490 (65.61)	
Yes	2658 (39.02)	984 (43.33)	893 (39.34)	781 (34.39)	
Cancer, *n* (%)					0.248
NO	6768 (99.35)	2261 (99.56)	2255 (99.34)	2252 (99.16)	
Yes	44 ( 0.65)	10 (0.44)	15 (0.66)	19 (0.84)	
CKD, *n* (%)					< 0.001
NO	6490 (95.27)	2207 (97.18)	2170 (95.59)	2113 (93.04)	
Yes	322 (4.73)	64 (2.82)	100 (4.41)	158 (6.96)	
FBG (mg/dL)	100.18 ± 10.92	99.93 ± 10.74	99.78 ± 10.85	100.83 ± 11.15	0.002
TC (mg/dL)	192.36 ± 37.00	189.61 ± 36.29	193.02 ± 37.29	194.44 ± 37.27	< 0.001
TG (mg/dL)	118.42 ± 73.67	115.07 ± 72.44	116.75 ± 71.38	123.45 ± 76.85	*<* 0.001
HDL-C (mg/dL)	52.36 ± 14.97	52.29 ± 14.97	52.92 ± 15.09	51.87 ± 14.83	0.057
LDL-C (mg/dL)	117.32 ± 33.52	115.59 ± 32.84	117.91 ± 34.02	118.46 ± 33.64	0.009
CRP, Median (IQR)	0.94 (0.52, 1.98)	0.85 (0.47, 1.73)	0.94 (0.54, 1.96)	1.07 (0.56, 2.29)	< 0.001
HGB (mg/dL)	14.37 ± 2.25	14.51 ± 2.15	14.44 ± 2.29	14.17 ± 2.30	< 0.001
CREA (mg/dL)	0.78 ± 0.24	0.78 ± 0.17	0.78 ± 0.18	0.77 ± 0.33	0.031
TyG, Mean ± SD	8.54 ± 0.54	8.51 ± 0.55	8.53 ± 0.54	8.60 ± 0.53	< 0.001
FI, Median (IQR)	0.12 (0.07, 0.20)	0.06 (0.03, 0.07)	0.12 (0.11, 0.15)	0.24 (0.20, 0.32)	< 0.001
CHG, Mean ± SD	5.23 ± 0.34	5.21 ± 0.34	5.22 ± 0.34	5.26 ± 0.35	< 0.001

IQR, Interquartile range; CKD, Chronic kidney disease; FBG, Fasting blood glucose; TC, Total cholesterol; TG, Triglycerides; HDL-C, High-density lipoprotein cholesterol; LDL-C, Low-density lipoprotein cholesterol; CRP, C-reactive protein; HGB, Hemoglobin; CREA, Creatinine; TyG, Triglyceride-glucose indices; FI, Frailty index; CHG, Cholesterol, high-density lipoprotein, and glucose indices; CHG-FI, Cholesterol, high-density lipoprotein, glucose, and frailty indices

### Correlation of CHG-FI with risk of heart disease, stroke, diabetes, and CMM

Table 2 shows results after full adjustment (Model 2). Each one-unit increment in CHG-FI was associated with higher risk for all outcomes. Heart disease: HR 1.34 (95% CI 1.23–1.45). Stroke: HR 1.85 (95% CI 1.65–2.06). Diabetes: HR 1.29 (95% CI 1.17–1.42). CMM: HR 1.79 (95% CI 1.58–2.04). In tertile-based analyses, participants in the highest tertile (T3) had substantially greater risks than those in the lowest tertile (T1) in the fully adjusted model (Model 2). The hazard ratios for T3 versus T1 were 2.00 (95% CI 1.72–2.32) for heart disease, 2.68 (95% CI 2.10–3.42) for stroke, 1.65 (95% CI 1.38–1.96) for diabetes, and 3.02 (95% CI 2.31–3.95) for CMM. All tests for trend were highly significant (*p* for trend < 0.001), indicating a clear dose–response relationship between higher CHG-FI and increased risk.

**Table 2:** Association between CHG-FI and risks of heart disease, stroke, diabetes, and CMM

Exposure	Outcome	Crude model		Model 1		Model 2	
		HR (95% CI)	*P* value	HR (95% CI)	*P* value	HR (95% CI)	*P* value
CHG-FI	Heart disease	1.46 (1.36 ~ 1.57)	< 0.001	1.35 (1.25 ~ 1.47)	< 0.001	1.34 (1.23 ~ 1.45)	< 0.001
*CHG-FI tertile*							
T1		1(Ref)		1(Ref)		1(Ref)	
T2		1.56 (1.34 ~ 1.81)	< 0.001	1.55 (1.33 ~ 1.80)	< 0.001	1.53 (1.32 ~ 1.79)	< 0.001
T3		2.19 (1.90 ~ 2.52)	< 0.001	2.05 (1.76 ~ 2.38)	< 0.001	2.00 (1.72 ~ 2.32)	< 0.001
P for trend			< 0.001		< 0.001		< 0.001
CHG-FI	Stroke	1.93 (1.75 ~ 2.13)	< 0.001	1.85 (1.66 ~ 2.07)	< 0.001	1.85 (1.65 ~ 2.06)	< 0.001
*CHG-FI tertile*							
T1		1(Ref)		1(Ref)		1(Ref)	
T2		1.94 (1.52 ~ 2.47)	< 0.001	1.88 (1.47 ~ 2.41)	< 0.001	1.87 (1.46 ~ 2.39)	< 0.001
T3		2.95 (2.34 ~ 3.72)	< 0.001	2.73 (2.14 ~ 3.48)	< 0.001	2.68 (2.10 ~ 3.42)	< 0.001
P for trend			< 0.001		< 0.001		< 0.001
CHG-FI	Diabetes	1.38 (1.26 ~ 1.50)	< 0.001	1.31 (1.19 ~ 1.45)	< 0.001	1.29 (1.17 ~ 1.42)	< 0.001
*CHG-FI tertile*							
T1		1(Ref)		1(Ref)		1(Ref)	
T2		1.40 (1.18 ~ 1.66)	< 0.001	1.36 (1.14 ~ 1.62)	0.001	1.34 (1.13 ~ 1.60)	0.001
T3		1.81 (1.53 ~ 2.13)	< 0.001	1.69 (1.42 ~ 2.01)	< 0.001	1.65 (1.38 ~ 1.96)	< 0.001
P for trend			< 0.001		< 0.001		< 0.001
CHG-FI	CMM	1.89 (1.69 ~ 2.12)	< 0.001	1.83 (1.62 ~ 2.07)	< 0.001	1.79 (1.58 ~ 2.04)	< 0.001
*CHG-FI tertile*							
T1		1(Ref)		1(Ref)		1(Ref)	
T2		1.86 (1.41 ~ 2.44)	< 0.001	1.84 (1.40 ~ 2.43)	< 0.001	1.81 (1.38 ~ 2.39)	< 0.001
T3		3.28 (2.55 ~ 4.22)	< 0.001	3.15 (2.41 ~ 4.11)	< 0.001	3.02 (2.31 ~ 3.95)	< 0.001
P for trend			< 0.001		< 0.001		< 0.001

Data presented are HRs and 95% CIs. Model 1: adjusted for age, sex, marital status, educational level, residence, smoking status, and drinking status. Model 2: adjusted for variables in Model 1 plus cancer, CKD, LDL-C, C-reactive protein, hemoglobin, and creatinine

HR, Hazard ratio; CI, Confidence interval; CHG-FI: Cholesterol, high-density lipoprotein, glucose and frailty indices; CMM: Cardiometabolic multimorbidity; CKD: Chronic kidney disease; LDL-C: Low-density lipoprotein cholesterol

**Fig. 2:**
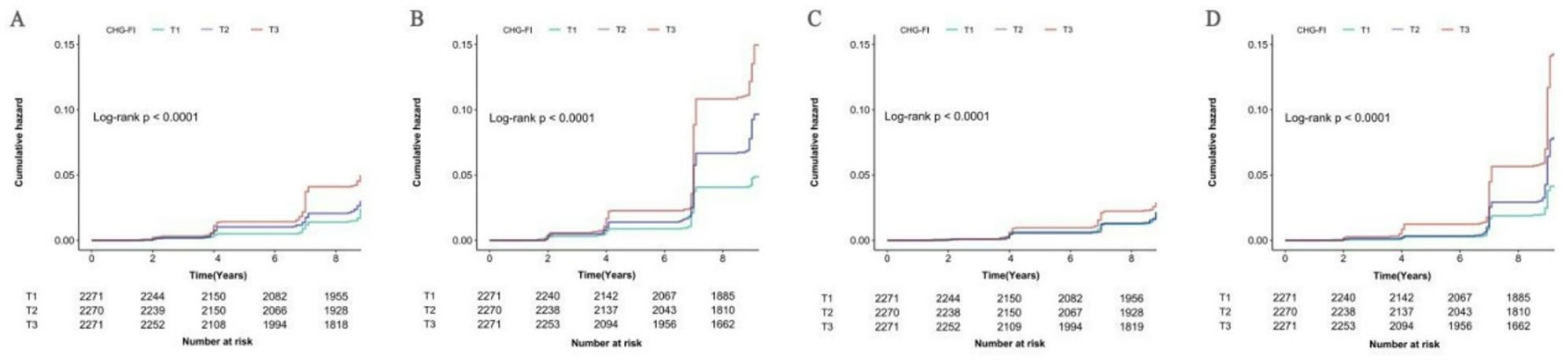
Kaplan-Meier survival curves for CHG-FI tertiles: heart disease (A), stroke (B), diabetes (C), and CMM (D). Abbreviations: CHG-FI: cholesterol, high-density lipoprotein, glucose and frailty indices; CMM: Cardiometabolic multimorbidity

**Fig. 3:**
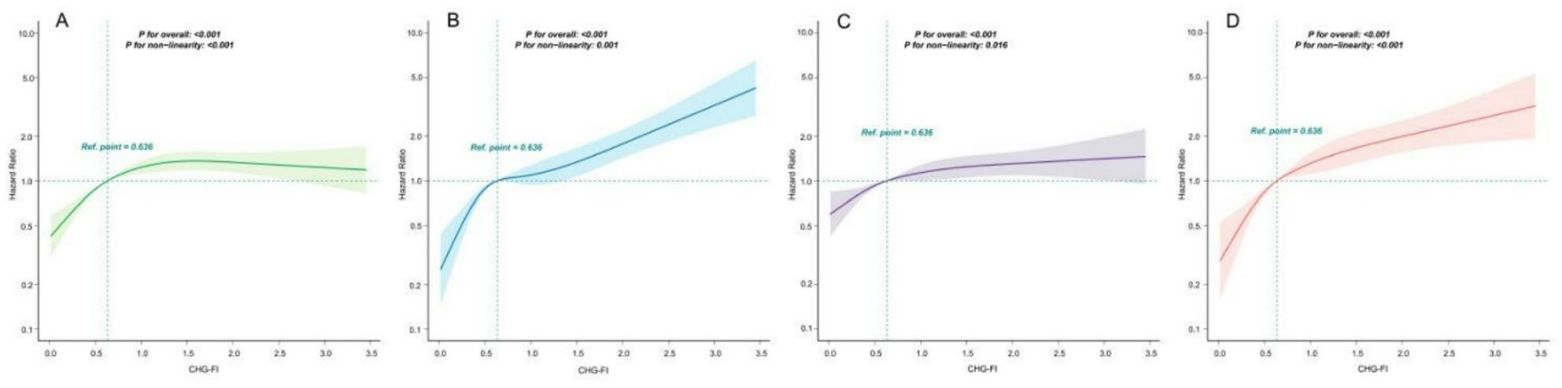
Dose-response associations between CHG-FI and heart disease (A), stroke (B), diabetes (C), and CMM (D) based on RCS models. Heavy central line represents the estimated adjusted hazard ratio, with shaded ribbons denoting 95% confidence interval. Reference point: Median. Models were adjusted for age, sex, marital status, educational level, residence, smoking status, drinking status, cancer, CKD, LDL-C, C-reactive protein, hemoglobin, and creatinine. Abbreviations: CHG-FI: cholesterol, high-density lipoprotein, glucose, and frailty indices; CMM: Cardiometabolic multimorbidity; CKD: chronic kidney disease; LDL-C: low-density lipoprotein cholesterol

Kaplan–Meier curves (Fig. 2) show a progressive rise in cumulative incidence from CHG-FI T1 to T3 for heart disease, stroke, diabetes, and CMM. Differences across tertiles were significant by log-rank test (*p* < 0.0001). Restricted cubic spline analyses (Fig. 3) revealed statistically significant non‑linear associations between CHG‑FI and each outcome (*p* for non‑linearity < 0.05). This pattern indicates an overall increasing association between CHG‑FI and outcome, but with detectable curvature (i.e., the increase is not perfectly linear across the entire CHG‑FI range).

### Interaction between CHG and FI

Table 3 and Supplementary Figure S1 present the interaction between CHG and FI on incident CMM after multi-variable adjustment. The multiplicative interaction term was 0.54 (95% CI 0.35–0.83, *P* = 0.005), indicating a statistically significant sub-multiplicative interaction. On the additive scale, RERI was − 0.24 (95% CI − 1.32 to 0.84), AP was − 0.05 (95% CI −0.29 to 0.18), and SI was 0.94 (95% CI 0.71–1.24); the confidence intervals include the null values (0 for RERI and AP, 1 for SI), indicating no statistically significant additive interaction.

**Table 3:** Multiplicative and additive interactions between CHG and FI on CMM risk

Measures	Estimates (95% CI)	*P*-value
*Multiplicative interaction*		
Multiplicative Scale	0.54 (0.35, 0.83)	0.005
*Additive interaction*		
RERI (95% CI)	−0.24 (−1.32, 0.84)	0.669
AP (95% CI)	−0.05 (−0.29, 0.18)	0.331
SI (95% CI)	0.94 (0.71,1.24)	0.262

Models were adjusted for age, sex, marital status, educational level, residence, smoking status, drinking status, history of cancer, CKD, LDL-C, C-reactive protein, hemoglobin, and creatinine. Estimates are reported as point estimates (95% CI); RERI, AP, and SI are also reported with 95% CIs

Interaction interpretation: for additive interaction, RERI = 0, AP = 0 or SI = 1 indicates no additive interaction; RERI > 0 or AP > 0 or SI > 1 indicates positive (synergistic) additive interaction (the reverse indicates antagonism). For the multiplicative scale, a value = 1 indicates no multiplicative interaction; >1 indicates positive multiplicative interaction; <1 indicates negative (antagonistic) multiplicative interaction

CHG, Cholesterol, high-density lipoprotein, and glucose indices; FI, Frailty index; CMM, Cardiometabolic multimorbidity; CKD, Chronic kidney disease; LDL-C, Low-density lipoprotein cholesterol. RERI, Relative Excess Risk Due to Interaction; AP, Attributable proportion; SI, synergy index

### Predictive value of CHG-FI for incident heart disease, stroke, diabetes, and CMM

**Fig. 4:**
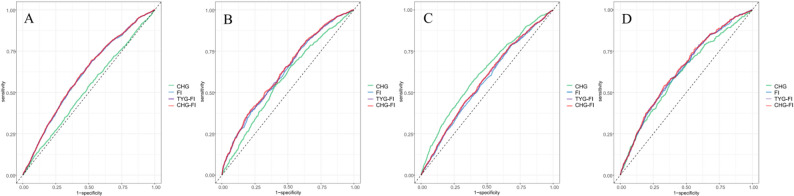
Receiver operating characteristic (ROC) curves for CHG‑FI predicting the risk of heart disease (A), stroke (B), diabetes (C), and CMM (D). Abbreviations: CHG: Cholesterol, high-density lipoprotein, glucose indices; FI: frailty index; TyG-FI: Triglyceride-glucose and frailty indices; CHG-FI: cholesterol, high-density lipoprotein, glucose and frailty indices; CMM: Cardiometabolic multimorbidity

Based on ROC curves (Fig. 4A–D) and corresponding ROC curve analysis values (Supplementary Table S15), CHG-FI showed modest but outcome-specific discriminatory performance. In conventional ROC analyses, CHG-FI reached the highest point estimates for stroke (AUC 63.02%) and CMM (AUC 63.80%), whereas for heart disease, CHG-FI, TyG-FI, and FI performed similarly (AUCs ≈ 60.40–60.64%) and CHG alone performed poorly (AUC 52.03%, 95% CI 50.27–53.78%). For diabetes, CHG alone had the highest AUC (61.91%, 95% CI 59.96–63.86%) while FI, TyG-FI, and CHG-FI were lower (≈ 56.36–57.47%). For CMM (Fig. 4D), CHG had an AUC of 61.24% (95% CI: 58.56–63.92%), and FI, TyG-FI, and CHG-FI showed incremental improvements (AUCs 62.81%, 63.49%, and 63.80%, respectively), with CHG-FI again highest. Sensitivity and specificity trade off across the indicators. For CMM, CHG-FI achieved the highest specificity (67.00%) .

Time-dependent ROC analyses (Supplementary Figure S2) corroborated these predictive patterns. The combined indices (CHG-FI and TyG-FI) exhibited superior and largely overlapping discriminative performance for CMM, with AUC values peaking at 0.70–0.72 during years 5–6, declining transiently to approximately 0.60 at year 7, and recovering to 0.63–0.65 by year 9. For stroke, these two combined indices remained closely aligned throughout the entire follow-up period (AUC ≈ 0.59–0.64), with only minimal divergence observed between the models. The FI alone demonstrated substantially poorer discriminative capacity for heart disease (AUC range: 0.45–0.52). In contrast, CHG as a single marker displayed early predictive superiority for diabetes, with an AUC of approximately 0.66 at year 4, followed by a gradual reduction to 0.57–0.62 in subsequent years. Collectively, the integration of metabolic indicators with the frailty index yielded moderate, outcome-specific, and time-dependent enhancements in prognostic accuracy, with the most pronounced incremental predictive value observed for CMM and heart disease.

Table 4 shows that adding FI, TyG-FI, or CHG-FI to baseline models produced modest but statistically significant gains in discrimination and consistent improvements in risk reclassification.

**Table 4:** Additional prognostic value of CHG-FI and associated measures

Model	AUC (95% CI)	*P*-value	NRI (95% CI)	*P*-value	IDI (95% CI)	*P*-value
*Heart disease*						
Basic model	0.588 (0.571 ~ 0.605)		Ref		Ref	
+TyG	0.590 (0.573 ~ 0.607)	0.416	0.092 (0.032 ~ 0.152)	0.003	0 (0 ~ 0.001)	0.046
+ CHG	0.589 (0.572 ~ 0.606)	0.752	0.036 (−0.025 ~ 0.096)	0.247	0 (0 ~ 0)	0.187
+ FI	0.622 (0.605 ~ 0.638)	< 0.001	0.225 (0.165 ~ 0.285)	< 0.001	0.009 (0.007 ~ 0.012)	< 0.001
+TyG-FI	0.623 (0.606 ~ 0.639)	< 0.001	0.223 (0.163 ~ 0.282)	< 0.001	0.009 (0.007 ~ 0.012)	< 0.001
+ CHG-FI	0.622 (0.606 ~ 0.639)	< 0.001	0.225 (0.165 ~ 0.285)	< 0.001	0.009 (0.007 ~ 0.012)	< 0.001
*Stroke*						
Basic model	0.609 (0.586 ~ 0.632)		Ref		Ref	
+TyG	0.623 (0.600 ~ 0.647)	0.033	0.192 (0.104 ~ 0.278)	< 0.001	0.004 (0.002 ~ 0.005)	< 0.001
+ CHG	0.629 (0.606 ~ 0.652)	0.011	0.234 (0.147 ~ 0.321)	< 0.001	0.005 (0.003 ~ 0.007)	< 0.001
+ FI	0.655 (0.633 ~ 0.678)	< 0.001	0.276 (0.189 ~ 0.362)	< 0.001	0.015 (0.010 ~ 0.019)	< 0.001
+TyG-FI	0.659 (0.636 ~ 0.681)	< 0.001	0.329 (0.236 ~ 0.409)	< 0.001	0.017 (0.012 ~ 0.021)	< 0.001
+ CHG-FI	0.660 (0.638 ~ 0.682)	< 0.001	0.310 (0.223 ~ 0.396)	< 0.001	0.017 (0.012 ~ 0.021)	< 0.001
*Diabetes*						
Basic model	0.573 (0.553 ~ 0.593)		Ref		Ref	
+TyG	0.618 (0.599 ~ 0.637)	< 0.001	0.312 (0.243 ~ 0.381)	< 0.001	0.015 (0.012 ~ 0.018)	< 0.001
+ CHG	0.637 (0.618 ~ 0.656)	< 0.001	0.326 (0.257 ~ 0.395)	< 0.001	0.023 (0.018 ~ 0.027)	< 0.001
+ FI	0.590 (0.570 ~ 0.610)	0.005	0.117 (0.049 ~ 0.186)	< 0.001	0.003 (0.002 ~ 0.005)	< 0.001
+ TyG-FI	0.594 (0.574 ~ 0.613)	0.001	0.141 (0.072 ~ 0.209)	< 0.001	0.005 (0.003 ~ 0.007)	< 0.001
+ CHG-FI	0.595 (0.575 ~ 0.614)	< 0.001	0.160 (0.091 ~ 0.228)	< 0.001	0.005 (0.003 ~ 0.007)	< 0.001
*CMM*						
Basic model	0.601 (0.574 ~ 0.628)		Ref		Ref	
+ TyG	0.630 (0.604 ~ 0.656)	0.003	0.300 (0.207 ~ 0.393)	< 0.001	0.006 (0.004 ~ 0.008)	< 0.001
+ CHG	0.644 (0.619 ~ 0.670)	< 0.001	0.301 (0.208 ~ 0.395)	< 0.001	0.009 (0.006 ~ 0.012)	< 0.001
+ FI	0.647 (0.621 ~ 0.673)	< 0.001	0.303 (0.209 ~ 0.397)	< 0.001	0.009 (0.006 ~ 0.013)	< 0.001
+ TyG-FI	0.652 (0.626 ~ 0.678)	< 0.001	0.328 (0.235 ~ 0.422)	< 0.001	0.011 (0.007 ~ 0.015)	< 0.001
+ CHG-FI	0.652 (0.627 ~ 0.678)	< 0.001	0.329 (0.236 ~ 0.423)	< 0.001	0.011 (0.008 ~ 0.015)	< 0.001

Basic models were adjusted for age, sex, marital status, educational level, residence, smoking status, drinking status, cancer, CKD, LDL-C, C-reactive protein, hemoglobin, and creatinine

AUC, Area Under the Curve; NRI, Net reclassification improvement; IDI, Integrated discrimination improvement; TyG, Triglyceride-Glucose Index; CHG, Cholesterol, high-density lipoprotein, glucose indices; FI, Frailty index; TyG-FI, Triglyceride-glucose and frailty indices; CHG-FI, Cholesterol, high-density lipoprotein, glucose and frailty indices; CMM, Cardiometabolic multimorbidity; CKD, Chronic kidney disease; LDL-C, Low-density lipoprotein cholesterol

For heart disease, the AUC increased from 0.588 to ~ 0.622–0.623 (*p* < 0.001), though TyG or CHG alone did not improve discrimination; NRI and IDI gains were primarily seen in FI-based models. For stroke and CMM, AUCs with CHG-FI increased to ~ 0.660 and ~ 0.652, respectively, with robust and concordant NRI/IDI improvements. For diabetes, the CHG index yielded the largest AUC increase (0.573→0.637), underscoring its stronger predictive value for diabetes. CHG-FI and TyG-FI provided broadly similar incremental performance.

We stratified the cohort by baseline triglyceride levels (TG < 150 mg/dL versus TG ≥ 150 mg/dL) and compared the predictive performance of TyG-FI and CHG-FI within each stratum. As shown in Supplementary Table S16, in the normotriglyceridemic subgroup (TG < 150 mg/dL), CHG-FI achieved robust predictive utility (AUC 0.651, NRI 0.364), performing equally to or marginally better than TyG-FI (AUC 0.649, NRI 0.346).

### Decision curve analysis of CHG-FI in predicting heart disease, stroke, diabetes and CMM

**Fig. 5:**
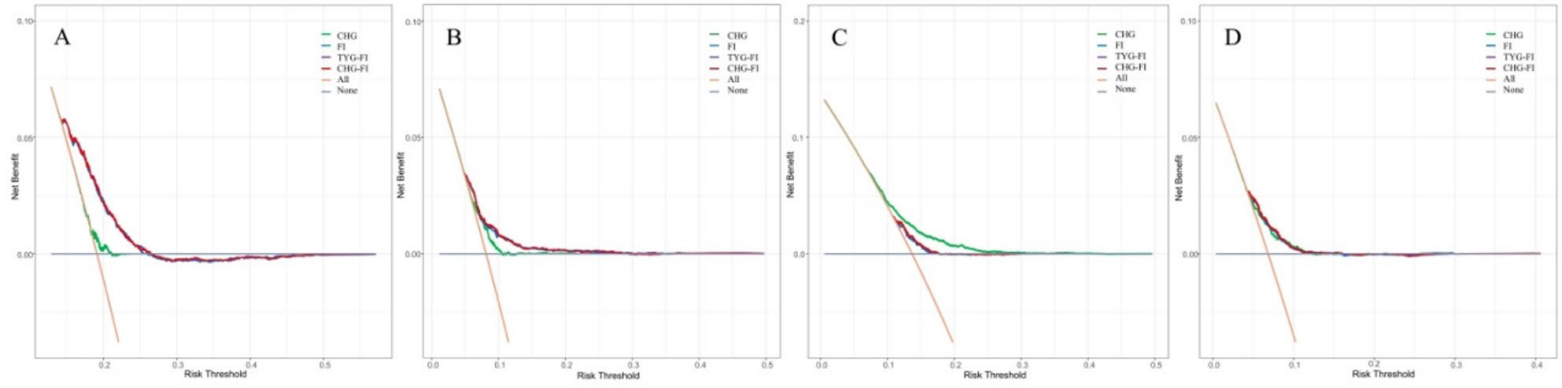
Decision curve analysis evaluating the net benefit of CHG‑FI versus other indices for predicting heart disease (panel A), stroke **B**, diabetes **C** and CMM **D**. CHG: Cholesterol, high-density lipoprotein, glucose indices; FI: frailty index; TyG-FI: Triglyceride-glucose and frailty indices; CHG-FI: cholesterol, high-density lipoprotein, glucose and frailty indices; CMM: Cardiometabolic multimorbidity

Decision curve analysis (Fig. 5) evaluated net clinical benefit across a range of risk thresholds. It used the treat-all and treat-none strategies as comparators. The combined CHG-FI and TyG-FI models, and FI, provided the largest net benefit across clinically relevant thresholds for incident heart disease, stroke, and CMM (Fig. 5A and B, and 5D). They outperformed the individual predictor CHG. By contrast, for incident diabetes (Fig. 5C), CHG alone provided the highest net benefit.

### Stratified associations of CHG‑FI with heart disease, stroke, diabetes, and CMM

**Fig. 6:**
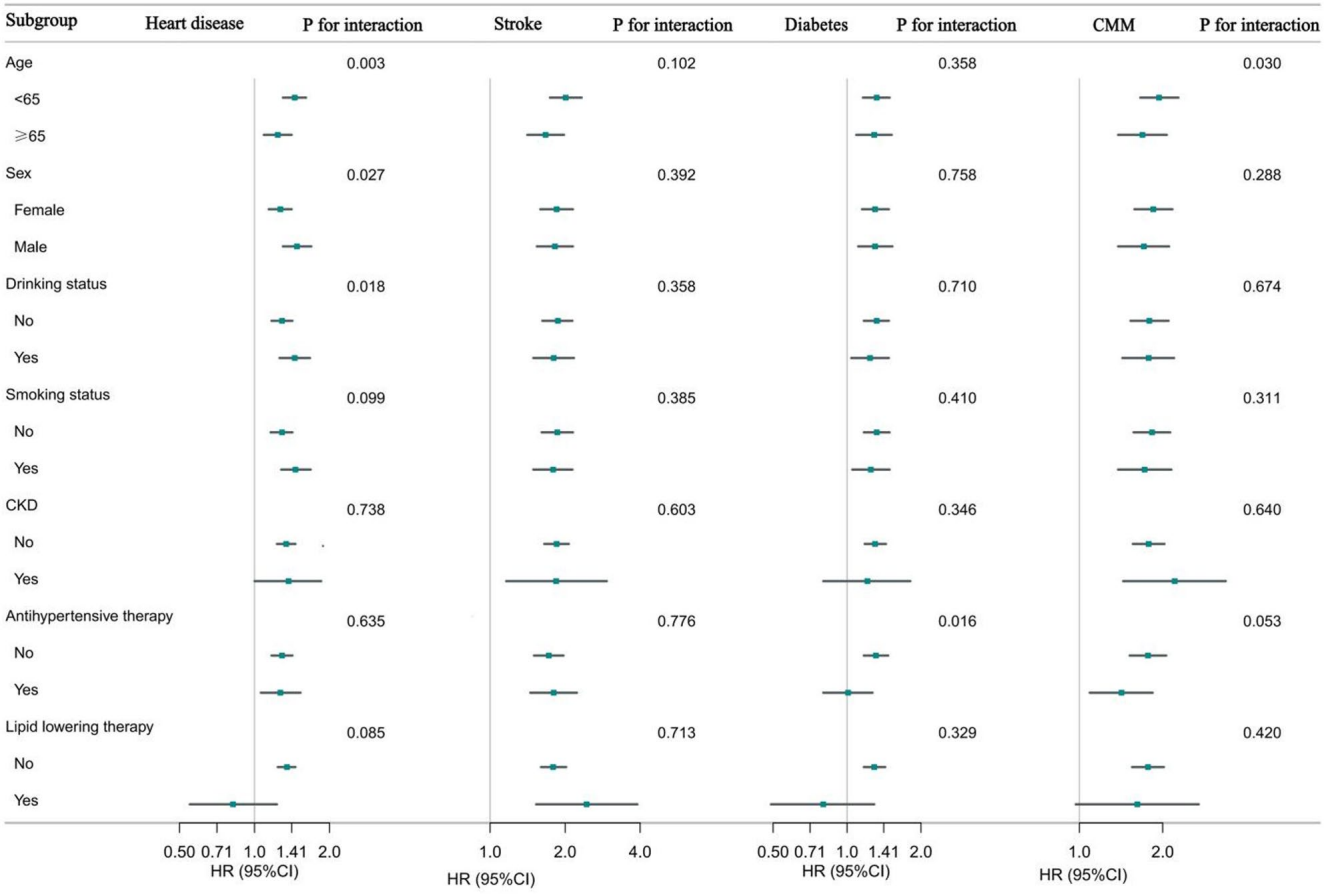
Stratified associations between CHG-FI and heart disease, stroke, diabetes, and CMM. All models were adjusted for age, sex, marital status, educational level, residence, smoking status, drinking status, cancer, CKD, LDL-C, C-reactive protein, hemoglobin, and creatinine. Abbreviations: CKD: chronic kidney disease; LDL-C: low-density lipoprotein cholesterol; CHG-FI: cholesterol, high-density lipoprotein, glucose and frailty indices; CMM: Cardiometabolic multimorbidity

Overall, subgroup interactions were mostly non‑significant. Interaction testing revealed that age, sex, and alcohol consumption significantly modified the association between CHG‑FI and incident heart disease. Age also significantly modified the association between CHG‑FI and incident CMM. The association with incident diabetes varied by antihypertensive treatment status. By contrast, no meaningful heterogeneity was observed for stroke across subgroups (Fig. 6).

### Sensitivity analyses

To assess robustness, we performed sensitivity analyses. First, results were consistent between the complete-case analysis and multiple-imputation datasets (Supplementary Table S5). Second, after excluding participants with baseline hypertension, cancer, or dyslipidemia, CHG-FI remained significantly associated with all four outcomes (Supplementary Table S6). Third, we excluded participants using antihypertensive agents, glucose-lowering, or lipid-lowering medications at baseline. The associations remained statistically robust (Supplementary Table S7). Fourth, to avoid circularity from including metabolic conditions in the Frailty Index, we removed hypertension from the 32‑item FI and recalculated the CHG‑FI. Results were similar (Supplementary Table S8). Fifth, we excluded self-reported histories of malignant tumors from the 32-item FI and recalculated the FI to avoid double counting cancer as both an FI component and a covariate; findings were consistent (Supplementary Table S9). Sixth, after adding body mass index (BMI) to the fully adjusted model, the associations remained unchanged (Supplementary Table S10). Seventh, applying an alternative definition of CKD based on self-report or estimated glomerular filtration rate (eGFR) < 60 mL/min/1.73 m² did not alter the results (Supplementary Table S11). Eighth, excluding participants who developed heart disease, stroke, diabetes, or CMM within the first two years of follow-up yielded similar findings (Supplementary Table S12). Ninth, accounting for the competing risk of death did not change the observed associations (Supplementary Table S13). Finally, E-values from tertile-based multi-variable models ranged from 2.69 to 5.49, supporting the robustness of the observed associations to unmeasured confounding (Supplementary Table S14).

## Discussion

In this large, nationally representative prospective cohort, we pioneered the application of a combined metabolic-frailty framework to evaluate the risk of incident CMM and its specific components. Rather than merely demonstrating longitudinal associations, our findings highlight the robust discriminative and reclassification value of the novel CHG-FI index. Elevated CHG-FI was significantly associated with non-linear, dose-response increases in the risk of heart disease, stroke, diabetes, and CMM. More importantly, the combined index yielded massive NRI and superior IDI compared to baseline models. A nuanced evaluation reveals a balanced predictive pattern: while the CHG index alone provided the strongest discrimination for incident diabetes, and FI alone was predominantly sufficient for predicting heart disease, the combined CHG-FI significantly outperformed singular indices in predicting complex, multi-system endpoints like stroke and CMM. This underscores the utility of CHG-FI not as a universal metric for all isolated diseases, but as a specific risk-stratification tool for end-stage multimorbidity.

Previous literature has validated the TyG index and its frailty combination (TyG-FI) as predictors of isolated cardiovascular events and stroke [21]. However, to date, no prior research has evaluated TyG-FI or any combined metabolic-frailty framework specifically for the incidence of CMM—a critical gap our study addresses. To benchmark our novel index, we conducted a rigorous head-to-head comparison. While CHG-FI and TyG-FI demonstrated predictive parity for CMM in the overall cohort (AUC 0.652 for both), this statistical equivalence masks crucial phenotypic divergence. TyG is heavily weighted toward triglycerides and glucose, capturing adiposity-related ectopic fat. In contrast, CHG combines the total cholesterol (TC)-to-HDL-C ratio with glucose, capturing cholesterol-mediated vascular burden. Crucially, our stratified analysis elucidated this advantage: among the “normotriglyceridemic” subgroup (TG < 150 mg/dL), CHG-FI achieved robust predictive utility (AUC 0.651, NRI 0.364), performing equally to or marginally better than TyG-FI (AUC 0.649, NRI 0.346). These findings suggest that CHG-FI may provide incremental diagnostic utility over TyG-FI specifically among older adults with normal triglyceride levels, a subgroup in whom atherosclerotic risk appears to be driven primarily by cholesterol–glucose metabolic pathways. Thus, CHG-FI emerges not as a redundant substitute, but as a mechanistically complementary alternative expanding the clinical toolkit.

The rationale for the combined CHG-FI index can be comprehensively interpreted through three distinct but interconnected layers: First, from a statistical perspective, our formal analysis revealed a significant interaction on the multiplicative scale between CHG and FI (HR 0.54, 95% CI 0.35–0.83, *P* = 0.01). This significant P-value mathematically mandates the use of a product term (CHG×FI) to accurately model the non-linear joint risk trajectory. However, the observed sub-multiplicative pattern (combined HR < 1.0) together with the absence of an additive interaction indicates that concurrent exposure to high metabolic burden and severe frailty produces a “risk saturation” or ceiling effect. Put differently, in severely frail individuals whose homeostatic reserves are markedly depleted, the marginal relative risk increment conferred by additional metabolic stress is smaller than that seen in more robust individuals. This is consistent with the results observed in previous studies [28, 29]. Second, from a biological perspective, we hypothesize that the link between CHG and FI is rooted in several converging pathological axes. A primary driver is “inflammaging”—a state of chronic, sterile, low-grade inflammation that characterizes both metabolic syndrome and biological aging [30, 31]. Dyslipidemia (cholesterol burden) and glucotoxicity (captured by CHG) stimulate the release of pro-inflammatory cytokines (e.g., CRP, IL-6), which in turn accelerate muscle wasting (sarcopenia) and cognitive decline, core components of the FI [32–35]. Furthermore, mitochondrial dysfunction serves as a critical cellular bridge. Metabolic stress impairs mitochondrial oxidative phosphorylation, leading to increased production of reactive oxygen species (ROS), which damages cellular proteins and DNA, thereby hastening the accumulation of physiological deficits represented by the FI [36, 37]. Finally, the cholesterol-glucose axis accelerates endothelial aging and arterial stiffness. depleting microvascular reserve and compromising host resilience to metabolic stress [38, 39]. Dyslipidemia and hyperglycaemia synergistically promote chronic inflammation, oxidative injury, and atherosclerosis. Concurrently, frailty denotes diminished physiological reserve and impaired compensatory capacity [40, 41]. Critically, endothelial dysfunction emerges as a convergent pathway: insulin resistance reduces nitric oxide bioavailability and accelerates vascular ageing, mechanisms that parallel cholesterol-glucose-mediated arterial damage [42–44]. This mechanistic overlap provides biological plausibility for a multiplicative interaction between CHG and FI. Future studies should elucidate mediating pathways through inflammatory markers (e.g., CRP, IL-6), muscle metabolism biomarkers (e.g., glutamine, creatine kinase), and endothelial function indices [45–47].

Regarding the interactive rationale, because metabolic dysfunction and frailty represent partially overlapping domains of a shared aging trajectory, combining them does not produce a biological “supra-additive” explosion, but rather captures the total cumulative load of vulnerability. Excluding either domain would leave the risk assessment incomplete. The incremental value observed in our NRI and IDI results confirms that measuring this cumulative load via CHG-FI yields significant practical utility for identifying high-risk phenotype, fully justifying its clinical application as an integrative risk-stratification tool.

This study offers several key strengths. First, it draws on a large, nationwide, representative prospective cohort with prolonged follow-up. Second, we applied rigorous statistical approaches, including formal interaction testing and competing-risk models, to enhance predictive utility. Third, multiple sensitivity analyses confirmed that the primary associations remained robust across alternative model specifications. Fourth, we successfully identified a phenotypically distinct risk stratification tool (CHG-FI) that provides significant net reclassification improvement over single markers for the primary outcome event CMM.

Several limitations temper our inferences. First, the observational design precludes causal attribution. Second, reliance on self-reported outcomes and health-deficit items introduces potential misclassification. Third, biomarkers were measured only at baseline, precluding analysis of temporal biomarker trajectories. Fourth, regarding the multiplicative interaction term (CHG × FI), we acknowledge three methodological limitations: the product term may confound main effects with interaction, the widely differing scales (CHG log-transformed; FI 0–1) disproportionately weight CHG, and clinical interpretability is somewhat diminished. We mitigated these concerns by utilizing tertile-based categories, analyzing components separately. Fifth, among 17,708 baseline participants, 6,812 (38%) were included in the final analysis after excluding those with missing data, prevalent CMM, or loss to follow-up. This substantial exclusion rate may have introduced selection bias. Consequently, findings may underestimate CMM risk in the general population and should be interpreted with caution when applied to broader demographics. Finally, the cohort is composed predominantly of middle-aged and older Chinese adults, limiting external validity.

## Conclusion

In conclusion, this study introduces the novel CHG-FI index, which integrates metabolic stress and biological aging to predict incident cardiometabolic multimorbidity. It improves risk reclassification for stroke and CMM, especially in normotriglyceridemic older adults, where TyG performs less well. Though showing sub-multiplicative interaction, CHG-FI captures cumulative vulnerability and supports multidimensional risk assessment for targeted prevention in aging populations.

## Electronic Supplementary Material

Below is the link to the electronic supplementary material.

Supplementary Material 1

## Data Availability

This study conducted an analysis of publicly available datasets. These data can be accessed here: https://charls.pku.edu.cn/.

## Abbreviations

ANOVA: Analysis of variance
AP: Attributable proportion
AUC: Area under the curve
CKD: Chronic kidney disease
CHARLS: China health and retirement longitudinal study
CHG: Cholesterol, High‑density Lipoprotein, Glucose Indices
CHG‑FI: Combined Cholesterol, High‑density Lipoprotein, Glucose and Frailty Indices
CI: Confidence interval
CMM: Cardiometabolic multimorbidity
CRP: C‑reactive protein
DCA: Decision curve analysis
FBG: Fasting blood glucose
FI: Frailty index
GVIF: Generalised variance inflation factor
HbA1c: Hemoglobin A1c
HDL‑C: High‑density lipoprotein cholesterol
HR: Hazard Ratio
IDI: Integrated discrimination improvement
IQR: Interquartile range
IRB: Institutional review board
LDL‑C: Low‑density lipoprotein cholesterol
NRI: Net reclassification improvement
PPS: Probability‑proportional‑to‑size
RERI: Relative excess risk due to interaction
ROC: Receiver operating characteristic
ROS: Reactive oxygen species
SD: Standard deviation
SI: Synergy index
TC: Total cholesterol
TG: Triglycerides
TyG: Triglyceride‑glucose indices
TyG‑FI: Triglyceride‑glucose and frailty indices
