# Supplementary material for: Predictive value of the combined cholesterol, high-density lipoprotein, glucose and frailty indices for cardiometabolic multimorbidity incidence: evidence from a national prospective cohort study
Source: Cardiovasc Diabetol. 2026 May 17;25:200. doi: 10.1186/s12933-026-03213-0 (PMC13348554; doi:10.1186/s12933-026-03213-0)
Supplement: Supplementary file 1 — Supplementary Material 1 [file 12933_2026_3213_MOESM1_ESM.docx]

**Supplementary Materials**

Contents

[Supplementary Table S1 Detailed description of the 32 items used to construct the frailty index in CHARLS and their corresponding cut-off values 1](#_Toc8557)

[Supplementary Table S2 Details of missing values 3](#_Toc6611)

[Supplementary Table S3 Collinearity Diagnostics for Covariates Based on Tolerance and Variance Inflation Factor(VIF) 4](#_Toc6078)

[Supplementary Table S4 Proportional hazards test for CHG-FI with heart disease, stroke, diabetes, and CMM 5](#_Toc20695)

[Supplementary Table S5 Excluding participants with missing covariate data in the overall sample 6](#_Toc5227)

[Supplementary Table S6 Exclusion of participants with baseline hypertension, cancer or 7](#_Toc9923)

[dyslipidaemia 7](#_Toc7022)

[Supplementary Table S7 Exclusion of participants using antihypertensive, 8](#_Toc12674)

[glucose‑lowering or lipid‑lowering medications 8](#_Toc28028)

[Supplementary Table S8 Remove hypertension from the 32-item Frailty Index and recalculate the CHG-FI 9](#_Toc28575)

[Supplementary Table S9 Remove cancer from the 32-item Frailty Index and recalculate 10](#_Toc29934)

[the CHG-FI 10](#_Toc30731)

[Supplementary Table S10 Associations between CHG-FI and the risks of heart disease, 11](#_Toc21258)

[stroke, diabetes, and CMM: Model 2 with BMI added 11](#_Toc31929)

[Supplementary Table S11 Associations between CHG-FI and risks of heart disease, stroke, 12](#_Toc22676)

[diabetes, and CMM using an alternative CKD definition 12](#_Toc24446)

[Supplementary Table S12 Excluding events occurring within the first two years of follow‑up 13](#_Toc19394)

[Supplementary Table S13 Competing-risk modeling was carried out using the Fine-Gray subdistribution hazards framework 14](#_Toc21147)

[Supplementary Table S14 E-values for the association between CHG-FI and heart disease, stroke, diabetes, and CMM 15](#_Toc12051)

[Supplementary Table S15 Receiver operating characteristic (ROC) curve analysis for CHG‑FI and related indices in predicting heart disease, stroke, diabetes, and CMM 15](#_Toc2768)

[Supplementary Table S16 Additional prognostic value of TyG-FI and CHG-FI for predicting CMM 16](#_Toc6040)

[Supplementary Figure S1 Interaction between CHG and FI in the development of new-onset CMM 17](#_Toc30456)

[Supplementary Figure S2 Time‑dependent predictive performance of CHG‑FI and related measures for incident outcomes: heart disease (A), stroke (B), diabetes (C) and CMM (D) across follow‑up years 4-9 18](#_Toc22179)

Supplementary Table S1 Detailed description of the 32 items used to construct the frailty index in CHARLS and their corresponding cut-off values

| No | Description of the items | Cut-off value |
| --- | --- | --- |
|  | CHARLS |  |
| 1 | Self-reported physician diagnosed hypertension | Yes = 1, No = 0 |
| 2 | Self-reported physician diagnosed diabetes | Yes = 1, No = 0 |
| 3 | Self-reported physician diagnosed heart disease |  |
| 4 | Self-reported physician diagnosed stroke |  |
| 5 | Self-reported physician diagnosed cancer | Yes = 1, No = 0 |
| 6 | Self-reported physician diagnosed arthritis | Yes = 1, No = 0 |
| 7 | Self-reported physician diagnosed chronic lung disease | Yes = 1, No = 0 |
| 8 | Self-reported physician diagnosed asthma | Yes = 1, No = 0 |
| 9 | Self-reported physician diagnosed any emotional, nervous, or psychiatric problems | Yes = 1, No = 0 |
| 10 | Self-reported physician diagnosed memory-related disease | Yes = 1, No = 0 |
| 11 | Self-reported vision problems | Yes = 1, No = 0 |
| 12 | Self-reported hearing problems | Yes = 1, No = 0 |
| 13 | Difficulty with dressing | Yes = 1, No = 0 |
| 14 | Difficulty with bathing or showering | Yes = 1, No = 0 |
| 15 | Difficulty with eating | Yes = 1, No = 0 |
| 16 | Difficulty with getting in and out of bed | Yes = 1, No = 0 |
| 17 | Difficulty with using the toilet | Yes = 1, No = 0 |
| 18 | Difficulty with managing money | Yes = 1, No = 0 |
| 19 | Difficulty with taking medications | Yes = 1, No = 0 |
| 20 | Difficulty with shopping for groceries | Yes = 1, No = 0 |
| 21 | Difficulty with preparing meals | Yes = 1, No = 0 |
| 22 | Difficulty with doing housework | Yes = 1, No = 0 |
| 23 | Mobility: difficulty with walking 100 yards | Yes = 1, No = 0 |
| 24 | Mobility: difficulty with getting up from a chair after sitting for long periods | Yes = 1, No = 0 |
| 25 | Mobility: difficulty with climbing several flights of stairs without resting | Yes = 1, No = 0 |
| 26 | Mobility: difficulty with lifting or carrying weights over 10 pounds/jins | Yes = 1, No = 0 |
| 27 | Mobility: difficulty with picking up a coin from the table | Yes = 1, No = 0 |
| 28 | Mobility: difficulty with stooping, kneeling, or crouching | Yes = 1, No = 0 |
| 29 | Mobility: difficulty with reaching arms above shoulder level | Yes = 1, No = 0 |
| 30 | Self-reported general health status | Poor or fair = 1, excellent, very good, or good = 0 |
| 31 | Depression: CESD-10 questionnaire | CESD-10 >10 =1, ≤10 =0 |
| 32 | Cognition: (memory test score + orientation test score) **/** 14 | Continuous, ranging from 0 to 1 |

Memory-related disease indicates Alzheimer’s disease or dementia, organic brain senility, or other serious memory impairment.

Depression is evaluated using Center for Epidemiologic Studies Depression Scale (CESD). In the CHARLS, CESD-10 is used, and the total score ranges from 0 to 30. The higher score indicates more severe depressive symptoms.

The memory score is the average of words that are not recalled in the immediate and delayed word recall tasks. The memory score ranges from 0 to 10. The orientation test comprises four questions about the day of the week, the month, the date of the month, and the year. One point is given for each wrong answer, and the range is from 0 to 4.

Supplementary Table S2 Details of missing values

| **Variable** | **Valid Samples** | **Missing Samples** | **Missing Proportion** |
| --- | --- | --- | --- |
| Age | 6812 | 0 | 0% |
| Sex | 6812 | 0 | 0% |
| Education level | 6812 | 5 | 0.1% |
| Marry | 6812 | 0 | 0% |
| Rural | 6812 | 0 | 0% |
| Smoking | 6812 | 1 | 0% |
| Drinking | 6812 | 6 | 0.1% |
| Cancer | 6812 | 9 | 0.1% |
| Kidney disease | 6812 | 17 | 0.2% |
| TG | 6812 | 0 | 0% |
| LDL-C | 6812 | 1 | 0% |
| CRP | 6812 | 0 | 0% |
| HGB | 6812 | 50 | 0.7% |
| Creatinine | 6812 | 0 | 0% |

Abbreviations:TG: triglycerides;LDL-C: low-density lipoprotein cholesterol; CRP: C-reactive protein; HGB:hemoglobin.

Supplementary Table S3 Collinearity Diagnostics for Covariates Based on Tolerance and Variance Inflation Factor(VIF)

| **Variables** | **GVIF** | **Df** | **GVIF^1/2Df^** |
| --- | --- | --- | --- |
| Age | 1.443 | 1 | 1.201 |
| Sex | 2.658 | 1 | 1.630 |
| Residence | 1.102 | 1 | 1.050 |
| Education level | 1.432 | 3 | 1.062 |
| Marital status | 1.156 | 1 | 1.075 |
| Smoking status | 2.034 | 1 | 1.426 |
| Drinking status | 1.426 | 1 | 1.194 |
| Cancer | 1.003 | 1 | 1.001 |
| CKD | 1.031 | 1 | 1.016 |
| HGB | 1.157 | 1 | 1.075 |
| Creatinine | 1.164 | 1 | 1.079 |
| CRP | 1.016 | 1 | 1.008 |
| LDL-C | 1.026 | 1 | 1.013 |

All adjusted GVIF^1/2Df^ were well below commonly used thresholds for concern (e.g., VIF≥ 2), indicating no meaningful multicollinearity among covariates. Accordingly, all listed variables were considered suitable for inclusion in subsequent multivariable analyses without further collinearity-driven adjustment.

Abbreviations:CKD: chronic kidney disease;HGB:hemoglobin; CRP: C-reactive protein;LDL-C: low-density lipoprotein cholesterol.

Supplementary Table S4 Proportional hazards test for CHG-FI with heart disease, stroke, diabetes, and CMM

| **Variable** | **Chi-square (χ²)** | **df** | ***P*-value** |
| --- | --- | --- | --- |
| **Heart diseas** |  |  |  |
| CHG-FI | 0.021 | 1 | 0.885 |
| GLOBAL | 0.021 | 1 | 0.885 |
| **Stroke** |  |  |  |
| CHG-FI | 0.901 | 1 | 0.343 |
| GLOBAL | 0.901 | 1 | 0.343 |
| **Diabetes** |  |  |  |
| CHG-FI | 2.784 | 1 | 0.095 |
| GLOBAL | 2.784 | 1 | 0.095 |
| **CMM** |  |  |  |
| CHG-FI | 0.459 | 1 | 0.498 |
| GLOBAL | 0.459 | 1 | 0.498 |

The proportional hazards assumption for Cox models including the CHG-FI was evaluated using the Schoenfeld-residuals approach (Grambsch–Therneau test), reporting chi-square statistics, degrees of freedom and two-sided P values. Tests were performed for each endpoint (heart disease, stroke, diabetes, and CMM) and a corresponding global test; a significance threshold of α=0.05 was used to identify departures from proportionality.All *P*-values exceeded 0.05, there was no evidence of violation of the proportional hazards assumption for CHG-FI in any model, supporting the validity of the Cox regression estimates reported.Abbreviations:CHG-FI:cholesterol, high-density lipoprotein,  glucose and frailty indices; CMM:Cardiometabolic multimorbidity.

Supplementary Table S5 Excluding participants with missing covariate data in the overall sample (n=6724)

| **Exposure** | **Outcome** | **Crude model** | | **Model 1** | | **Model 2** | |
| --- | --- | --- | --- | --- | --- | --- | --- |
|  |  | **HR(95% CI)** | ***P*-value** | **HR(95% CI)** | ***P*-value** | **HR(95% CI)** | ***P*-value** |
| CHG-FI | Heart disease | 1.46 (1.35~1.57) | <0.001 | 1.35 (1.24~1.46) | <0.001 | 1.33 (1.22~1.44) | <0.001 |
| CHG-FI tertile |  |  |  |  |  |  |  |
| T1 |  | 1(Ref) |  | 1(Ref) |  | 1(Ref) |  |
| T2 |  | 1.55 (1.33~1.8) | <0.001 | 1.53 (1.32~1.79) | <0.001 | 1.52 (1.3~1.77) | <0.001 |
| T3 |  | 2.17 (1.88~2.5) | <0.001 | 2.03 (1.74~2.36) | <0.001 | 1.98 (1.7~2.31) | <0.001 |
| P for trend |  |  | <0.001 |  | <0.001 |  | <0.001 |
| CHG-FI | Stroke | 1.93 (1.74~2.13) | <0.001 | 1.85 (1.65~2.06) | <0.001 | 1.84 (1.64~2.06) | <0.001 |
| CHG-FI tertile |  |  |  |  |  |  |  |
| T1 |  | 1(Ref) |  | 1(Ref) |  | 1(Ref) |  |
| T2 |  | 1.9 (1.49~2.43) | <0.001 | 1.85 (1.44~2.36) | <0.001 | 1.83 (1.43~2.34) | <0.001 |
| T3 |  | 2.91 (2.31~3.66) | <0.001 | 2.69 (2.11~3.42) | <0.001 | 2.64 (2.07~3.37) | <0.001 |
| P for trend |  |  | <0.001 |  | <0.001 |  | <0.001 |
| CHG-FI | Diabetes | 1.38 (1.26~1.51) | <0.001 | 1.32 (1.19~1.45) | <0.001 | 1.29 (1.17~1.43) | <0.001 |
| CHG-FI tertile |  |  |  |  |  |  |  |
| T1 |  | 1(Ref) |  | 1(Ref) |  | 1(Ref) |  |
| T2 |  | 1.4 (1.18~1.67) | <0.001 | 1.36 (1.14~1.62) | 0.001 | 1.34 (1.13~1.6) | 0.001 |
| T3 |  | 1.84 (1.56~2.17) | <0.001 | 1.72 (1.45~2.05) | <0.001 | 1.68 (1.41~2) | <0.001 |
| P for trend |  |  | <0.001 |  | <0.001 |  | <0.001 |
| CHG-FI | CMM | 1.89 (1.69~2.12) | <0.001 | 1.82 (1.61~2.06) | <0.001 | 1.78 (1.57~2.03) | <0.001 |
| CHG-FI tertile |  |  |  |  |  |  |  |
| T1 |  | 1(Ref) |  | 1(Ref) |  | 1(Ref) |  |
| T2 |  | 1.83 (1.39~2.4) | <0.001 | 1.8 (1.37~2.38) | <0.001 | 1.77 (1.35~2.34) | <0.001 |
| T3 |  | 3.26 (2.53~4.2) | <0.001 | 3.11 (2.39~4.06) | <0.001 | 2.98 (2.28~3.9) | <0.001 |
| P for trend |  |  | <0.001 |  | <0.001 |  | <0.001 |

Data presented are HRs and 95% CIs.Model 1: adjusted for age, sex, marital status, educational level, residence, smoking status and drinking status.Model 2: adjusted for variables in Model 1 plus cancer,CKD,LDL-C, C-reactive protein,hemoglobin,and creatinine. Abbreviations:HR, hazard ratio; CI, confidence interval; Ref, reference;CKD: chronic kidney disease;LDL-C: low-density lipoprotein cholesterol;CHG-FI:cholesterol, high-density lipoprotein,  glucose and frailty indices; CMM:Cardiometabolic multimorbidity.

Supplementary Table S6 Exclusion of participants with baseline hypertension, cancer or

dyslipidaemia（n=4197)

| **Exposure** | **Outcome** | **Crude model** | | **Model 1** | | **Model 2** | |
| --- | --- | --- | --- | --- | --- | --- | --- |
|  |  | **HR(95% CI)** | ***P*-value** | **HR(95% CI)** | ***P*-value** | **HR(95% CI)** | ***P*-value** |
| CHG-FI | Heart disease | 1.65 (1.49~1.84) | <0.001 | 1.47 (1.31~1.65) | <0.001 | 1.45 (1.29~1.64) | <0.001 |
| CHG-FI tertile |  |  |  |  |  |  |  |
| T1 |  | 1(Ref) |  | 1(Ref) |  | 1(Ref) |  |
| T2 |  | 1.47 (1.19~1.81) | <0.001 | 1.48 (1.2~1.84) | <0.001 | 1.48 (1.2~1.83) | <0.001 |
| T3 |  | 2.25 (1.85~2.73) | <0.001 | 2.09 (1.7~2.57) | <0.001 | 2.05 (1.66~2.52) | <0.001 |
| P for trend |  |  | <0.001 |  | <0.001 |  | <0.001 |
| CHG-FI | Stroke | 2.24 (1.91~2.62) | <0.001 | 1.99 (1.67~2.37) | <0.001 | 1.99 (1.67~2.38) | <0.001 |
| CHG-FI tertile |  |  |  |  |  |  |  |
| T1 |  | 1(Ref) |  | 1(Ref) |  | 1(Ref) |  |
| T2 |  | 2.21 (1.48~3.3) | <0.001 | 2.08 (1.39~3.12) | <0.001 | 2.09 (1.39~3.14) | <0.001 |
| T3 |  | 3.82 (2.63~5.55) | <0.001 | 3.22 (2.17~4.77) | <0.001 | 3.23 (2.17~4.79) | <0.001 |
| P for trend |  |  | <0.001 |  | <0.001 |  | <0.001 |
| CHG-FI | Diabetes | 1.38 (1.2~1.58) | <0.001 | 1.28 (1.1~1.49) | 0.002 | 1.25 (1.07~1.46) | 0.005 |
| CHG-FI tertile |  |  |  |  |  |  |  |
| T1 |  | 1(Ref) |  | 1(Ref) |  | 1(Ref) |  |
| T2 |  | 1.49 (1.17~1.9) | 0.001 | 1.44 (1.13~1.84) | 0.004 | 1.44 (1.13~1.84) | 0.003 |
| T3 |  | 1.79 (1.42~2.26) | <0.001 | 1.64 (1.28~2.09) | <0.001 | 1.6 (1.25~2.05) | <0.001 |
| P for trend |  |  | <0.001 |  | <0.001 |  | <0.001 |
| CHG-FI | CMM | 2.32 (1.94~2.77) | <0.001 | 2.11 (1.73~2.58) | <0.001 | 2.03 (1.66~2.5) | <0.001 |
| CHG-FI tertile |  |  |  |  |  |  |  |
| T1 |  | 1(Ref) |  | 1(Ref) |  | 1(Ref) |  |
| T2 |  | 2.1 (1.32~3.33) | 0.002 | 2.06 (1.29~3.29) | 0.002 | 2.04 (1.28~3.26) | 0.003 |
| T3 |  | 4 (2.61~6.11) | <0.001 | 3.58 (2.29~5.6) | <0.001 | 3.39 (2.16~5.32) | <0.001 |
| P for trend |  |  | <0.001 |  | <0.001 |  | <0.001 |

Data presented are HRs and 95% CIs.Model 1: adjusted for age, sex, marital status, educational level, residence, smoking status and drinking status.Model 2: adjusted for variables in Model 1 plus cancer,CKD,LDL-C, C-reactive protein,hemoglobin,and creatinine. Abbreviations:HR, hazard ratio; CI, confidence interval; Ref, reference;CKD: chronic kidney disease;LDL-C: low-density lipoprotein cholesterol;CHG-FI:cholesterol, high-density lipoprotein,  glucose and frailty indices; CMM:Cardiometabolic multimorbidity.

Supplementary Table S7 Exclusion of participants using antihypertensive, glucose‑lowering

or lipid‑lowering medications(n=5767)

| **Exposure** | **Outcome** | **Crude model** | | **Model 1** | | **Model 2** | |
| --- | --- | --- | --- | --- | --- | --- | --- |
|  |  | **HR(95% CI)** | ***P*-value** | **HR(95% CI)** | ***P*-value** | **HR(95% CI)** | ***P*-value** |
| CHG-FI | Heart disease | 1.45 (1.33~1.58) | <0.001 | 1.32 (1.21~1.45) | <0.001 | 1.3 (1.18~1.43) | <0.001 |
| CHG-FI tertile |  |  |  |  |  |  |  |
| T1 |  | 1(Ref) |  | 1(Ref) |  | 1(Ref) |  |
| T2 |  | 1.43 (1.2~1.7) | <0.001 | 1.43 (1.2~1.7) | <0.001 | 1.42 (1.19~1.68) | <0.001 |
| T3 |  | 2.06 (1.76~2.42) | <0.001 | 1.95 (1.64~2.31) | <0.001 | 1.9 (1.6~2.25) | <0.001 |
| P for trend |  |  | <0.001 |  | <0.001 |  | <0.001 |
| CHG-FI | Stroke | 1.88 (1.66~2.12) | <0.001 | 1.73 (1.51~1.99) | <0.001 | 1.73 (1.51~1.99) | <0.001 |
| CHG-FI tertile |  |  |  |  |  |  |  |
| T1 |  | 1(Ref) |  | 1(Ref) |  | 1(Ref) |  |
| T2 |  | 1.68 (1.27~2.23) | <0.001 | 1.6 (1.2~2.12) | 0.001 | 1.61 (1.21~2.14) | 0.001 |
| T3 |  | 2.5 (1.92~3.26) | <0.001 | 2.22 (1.68~2.93) | <0.001 | 2.21 (1.67~2.92) | <0.001 |
| P for trend |  |  | <0.001 |  | <0.001 |  | <0.001 |
| CHG-FI | Diabetes | 1.41 (1.28~1.57) | <0.001 | 1.35 (1.2~1.51) | <0.001 | 1.32 (1.18~1.49) | <0.001 |
| CHG-FI tertile |  |  |  |  |  |  |  |
| T1 |  | 1(Ref) |  | 1(Ref) |  | 1(Ref) |  |
| T2 |  | 1.47 (1.21~1.79) | <0.001 | 1.42 (1.17~1.73) | 0.001 | 1.41 (1.16~1.73) | 0.001 |
| T3 |  | 1.76 (1.46~2.13) | <0.001 | 1.63 (1.34~2) | <0.001 | 1.6 (1.31~1.96) | <0.001 |
| P for trend |  |  | <0.001 |  | <0.001 |  | <0.001 |
| CHG-FI | CMM | 1.97 (1.72~2.25) | <0.001 | 1.86 (1.6~2.16) | <0.001 | 1.81 (1.55~2.11) | <0.001 |
| CHG-FI tertile |  |  |  |  |  |  |  |
| T1 |  | 1(Ref) |  | 1(Ref) |  | 1(Ref) |  |
| T2 |  | 1.92 (1.38~2.67) | <0.001 | 1.88 (1.34~2.62) | <0.001 | 1.86 (1.33~2.6) | <0.001 |
| T3 |  | 3.26 (2.4~4.43) | <0.001 | 3.06 (2.22~4.23) | <0.001 | 2.93 (2.12~4.05) | <0.001 |
| P for trend |  |  | <0.001 |  | <0.001 |  | <0.001 |

Data presented are HRs and 95% CIs.Model 1: adjusted for age, sex, marital status, educational level, residence, smoking status and drinking status.Model 2: adjusted for variables in Model 1 plus cancer,CKD,LDL-C, C-reactive protein,hemoglobin,and creatinine. Abbreviations:HR, hazard ratio; CI, confidence interval; Ref, reference;CKD: chronic kidney disease;LDL-C: low-density lipoprotein cholesterol;CHG-FI:cholesterol, high-density lipoprotein,  glucose and frailty indices; CMM:Cardiometabolic multimorbidity.

Supplementary Table S8 Remove hypertension from the 32-item Frailty Index and recalculate the CHG-FI(n=6812)

| **Exposure** | **Outcome** | **Crude model** | | **Model 1** | | **Model 2** | |
| --- | --- | --- | --- | --- | --- | --- | --- |
|  |  | **HR(95% CI)** | ***P*-value** | **HR(95% CI)** | ***P*-value** | **HR(95% CI)** | ***P*-value** |
| CHG-FI | Heart disease | 1.41 (1.32~1.52) | <0.001 | 1.31 (1.21~1.42) | <0.001 | 1.29 (1.19~1.4) | <0.001 |
| CHG-FI tertile |  |  |  |  |  |  |  |
| T1 |  | 1(Ref) |  | 1(Ref) |  | 1(Ref) |  |
| T2 |  | 1.43 (1.24~1.66) | <0.001 | 1.44 (1.24~1.67) | <0.001 | 1.43 (1.23~1.66) | <0.001 |
| T3 |  | 1.97 (1.72~2.27) | <0.001 | 1.87 (1.61~2.17) | <0.001 | 1.83 (1.58~2.13) | <0.001 |
| P for trend |  |  | <0.001 |  | <0.001 |  | <0.001 |
| CHG-FI | Stroke | 1.82 (1.65~2.01) | <0.001 | 1.73 (1.55~1.94) | <0.001 | 1.73 (1.55~1.93) | <0.001 |
| CHG-FI tertile |  |  |  |  |  |  |  |
| T1 |  | 1(Ref) |  | 1(Ref) |  | 1(Ref) |  |
| T2 |  | 1.45 (1.14~1.83) | 0.002 | 1.39 (1.1~1.76) | 0.006 | 1.4 (1.1~1.77) | 0.006 |
| T3 |  | 2.35 (1.89~2.91) | <0.001 | 2.16 (1.72~2.72) | <0.001 | 2.15 (1.71~2.71) | <0.001 |
| P for trend |  |  | <0.001 |  | <0.001 |  | <0.001 |
| CHG-FI | Diabetes | 1.32 (1.2~1.44) | <0.001 | 1.25 (1.13~1.38) | <0.001 | 1.23 (1.11~1.36) | <0.001 |
| CHG-FI tertile |  |  |  |  |  |  |  |
| T1 |  | 1(Ref) |  | 1(Ref) |  | 1(Ref) |  |
| T2 |  | 1.34 (1.13~1.58) | 0.001 | 1.29 (1.09~1.53) | 0.004 | 1.29 (1.08~1.53) | 0.004 |
| T3 |  | 1.61 (1.37~1.89) | <0.001 | 1.49 (1.25~1.77) | <0.001 | 1.46 (1.23~1.74) | <0.001 |
| P for trend |  |  | <0.001 |  | <0.001 |  | <0.001 |
| CHG-FI | CMM | 1.77 (1.58~1.98) | <0.001 | 1.69 (1.5~1.92) | <0.001 | 1.66 (1.47~1.89) | <0.001 |
| CHG-FI tertile |  |  |  |  |  |  |  |
| T1 |  | 1(Ref) |  | 1(Ref) |  | 1(Ref) |  |
| T2 |  | 1.52 (1.17~1.97) | 0.002 | 1.5 (1.15~1.95) | 0.003 | 1.49 (1.15~1.95) | 0.003 |
| T3 |  | 2.62 (2.06~3.33) | <0.001 | 2.51 (1.94~3.23) | <0.001 | 2.43 (1.88~3.13) | <0.001 |
| P for trend |  |  | <0.001 |  | <0.001 |  | <0.001 |

Data presented are HRs and 95% CIs.Model 1: adjusted for age, sex, marital status, educational level, residence, smoking status and drinking status.Model 2: adjusted for variables in Model 1 plus cancer,CKD,LDL-C, C-reactive protein,hemoglobin,and creatinine. Abbreviations:HR, hazard ratio; CI, confidence interval; Ref, reference;CKD: chronic kidney disease;LDL-C: low-density lipoprotein cholesterol;CHG-FI:cholesterol, high-density lipoprotein,  glucose and frailty indices; CMM:Cardiometabolic multimorbidity.

Supplementary Table S9 Remove cancer from the 32-item Frailty Index and recalculate

the CHG-FI(n=6812)

| **Exposure** | **Outcome** | **Crude model** | | **Model 1** | | **Model 2** | |
| --- | --- | --- | --- | --- | --- | --- | --- |
|  |  | **HR**  **(95% CI)** | ***P*-value** | **HR**  **(95% CI)** | ***P*-value** | **HR**  **(95% CI)** | ***P*-value** |
| CHG-FI | Heart disease | 1.45 (1.35~1.55) | <0.001 | 1.34 (1.24~1.45) | <0.001 | 1.33 (1.23~1.43) | <0.001 |
| CHG-FI tertile |  |  |  |  |  |  |  |
| T1 |  | 1(Ref) |  | 1(Ref) |  | 1(Ref) |  |
| T2 |  | 1.55 (1.34~1.8) | <0.001 | 1.54 (1.32~1.79) | <0.001 | 1.53 (1.31~1.78) | <0.001 |
| T3 |  | 2.17 (1.88~2.5) | <0.001 | 2.03 (1.75~2.36) | <0.001 | 1.98 (1.71~2.31) | <0.001 |
| P for trend |  |  | <0.001 |  | <0.001 |  | <0.001 |
| CHG-FI | Stroke | 1.89 (1.72~2.08) | <0.001 | 1.82 (1.63~2.02) | <0.001 | 1.81 (1.63~2.01) | <0.001 |
| CHG-FI tertile |  |  |  |  |  |  |  |
| T1 |  | 1(Ref) |  | 1(Ref) |  | 1(Ref) |  |
| T2 |  | 1.94 (1.52~2.48) | <0.001 | 1.89 (1.48~2.42) | <0.001 | 1.87 (1.46~2.4) | <0.001 |
| T3 |  | 3 (2.38~3.79) | <0.001 | 2.78 (2.18~3.55) | <0.001 | 2.73 (2.14~3.48) | <0.001 |
| P for trend |  |  | <0.001 |  | <0.001 |  | <0.001 |
| CHG-FI | Diabetes | 1.36 (1.25~1.48) | <0.001 | 1.3 (1.18~1.43) | <0.001 | 1.28 (1.16~1.41) | <0.001 |
| CHG-FI tertile |  |  |  |  |  |  |  |
| T1 |  | 1(Ref) |  | 1(Ref) |  | 1(Ref) |  |
| T2 |  | 1.41 (1.18~1.67) | <0.001 | 1.37 (1.15~1.63) | <0.001 | 1.35 (1.14~1.61) | 0.001 |
| T3 |  | 1.81 (1.54~2.13) | <0.001 | 1.69 (1.42~2.01) | <0.001 | 1.65 (1.38~1.96) | <0.001 |
| P for trend |  |  | <0.001 |  | <0.001 |  | <0.001 |
| CHG-FI | CMM | 1.86 (1.67~2.07) | <0.001 | 1.79 (1.59~2.02) | <0.001 | 1.76 (1.56~1.99) | <0.001 |
| CHG-FI tertile |  |  |  |  |  |  |  |
| T1 |  | 1(Ref) |  | 1(Ref) |  | 1(Ref) |  |
| T2 |  | 1.85 (1.41~2.43) | <0.001 | 1.84 (1.4~2.42) | <0.001 | 1.81 (1.37~2.38) | <0.001 |
| T3 |  | 3.28 (2.55~4.22) | <0.001 | 3.15 (2.42~4.11) | <0.001 | 3.02 (2.31~3.94) | <0.001 |
| P for trend |  |  | <0.001 |  | <0.001 |  | <0.001 |

Data presented are HRs and 95% CIs.Model 1: adjusted for age, sex, marital status, educational level, residence, smoking status and drinking status.Model 2: adjusted for variables in Model 1 plus cancer,CKD,LDL-C, C-reactive protein,hemoglobin,and creatinine. Abbreviations:HR, hazard ratio; CI, confidence interval; Ref, reference;CKD: chronic kidney disease;LDL-C: low-density lipoprotein cholesterol;CHG-FI:cholesterol, high-density lipoprotein,  glucose and frailty indices; CMM:Cardiometabolic multimorbidity.

Supplementary Table S10 Associations between CHG-FI and the risks of heart disease,

stroke, diabetes, and CMM: Model 2 with BMI added (n = 6,812)

| **Exposure** | **Outcome** | **Crude model** | | **Model 1** | | **Model 2** | |
| --- | --- | --- | --- | --- | --- | --- | --- |
|  |  | **HR**  **(95% CI)** | ***P*-value** | **HR**  **(95% CI)** | ***P*-value** | **HR**  **(95% CI)** | ***P*-value** |
| CHG-FI | Heart disease | 1.46 (1.36~1.57) | <0.001 | 1.35 (1.25~1.47) | <0.001 | 1.34 (1.23~1.45) | <0.001 |
| CHG-FI tertile |  |  |  |  |  |  |  |
| T1 |  | 1(Ref) |  | 1(Ref) |  | 1(Ref) |  |
| T2 |  | 1.56 (1.34~1.81) | <0.001 | 1.55 (1.33~1.8) | <0.001 | 1.53 (1.32~1.79) | <0.001 |
| T3 |  | 2.19 (1.9~2.52) | <0.001 | 2.05 (1.76~2.38) | <0.001 | 2 (1.72~2.32) | <0.001 |
| P for trend |  |  | <0.001 |  | <0.001 |  | <0.001 |
| CHG-FI | Stroke | 1.93 (1.75~2.13) | <0.001 | 1.85 (1.66~2.07) | <0.001 | 1.85 (1.65~2.06) | <0.001 |
| CHG-FI tertile |  |  |  |  |  |  |  |
| T1 |  | 1(Ref) |  | 1(Ref) |  | 1(Ref) |  |
| T2 |  | 1.94 (1.52~2.47) | <0.001 | 1.88 (1.47~2.41) | <0.001 | 1.87 (1.46~2.39) | <0.001 |
| T3 |  | 2.95 (2.34~3.72) | <0.001 | 2.73 (2.14~3.48) | <0.001 | 2.68 (2.1~3.42) | <0.001 |
| P for trend |  |  | <0.001 |  | <0.001 |  | <0.001 |
| CHG-FI | Diabetes | 1.38 (1.26~1.5) | <0.001 | 1.31 (1.19~1.45) | <0.001 | 1.29 (1.17~1.42) | <0.001 |
| CHG-FI tertile |  |  |  |  |  |  |  |
| T1 |  | 1(Ref) |  | 1(Ref) |  | 1(Ref) |  |
| T2 |  | 1.4 (1.18~1.66) | <0.001 | 1.36 (1.14~1.62) | 0.001 | 1.34 (1.13~1.6) | 0.001 |
| T3 |  | 1.81 (1.53~2.13) | <0.001 | 1.69 (1.42~2.01) | <0.001 | 1.65 (1.38~1.96) | <0.001 |
| P for trend |  |  | <0.001 |  | <0.001 | ) | <0.001 |
| CHG-FI | CMM | 1.89 (1.69~2.12) | <0.001 | 1.83 (1.62~2.07) | <0.001 | 1.79 (1.58~2.04) | <0.001 |
| CHG-FI tertile |  |  |  |  |  |  |  |
| T1 |  | 1(Ref) |  | 1(Ref) |  | 1(Ref) |  |
| T2 |  | 1.86 (1.41~2.44) | <0.001 | 1.84 (1.4~2.43) | <0.001 | 1.81 (1.38~2.39) | <0.001 |
| T3 |  | 3.28 (2.55~4.22) | <0.001 | 3.15 (2.41~4.11) | <0.001 | 3.02 (2.31~3.95) | <0.001 |
| P for trend |  |  | <0.001 |  | <0.001 |  | <0.001 |

Data presented are HRs and 95% CIs.Model 1: adjusted for age, sex, marital status, educational level, residence, smoking status and drinking status.Model 2: adjusted for variables in Model 1 plus BMI,cancer,CKD,LDL-C, C-reactive protein,hemoglobin,and creatinine. Abbreviations:HR, hazard ratio; CI, confidence interval; Ref, reference;CKD: chronic kidney disease;LDL-C: low-density lipoprotein cholesterol;CHG-FI:cholesterol, high-density lipoprotein,  glucose and frailty indices; CMM:Cardiometabolic multimorbidity.

Supplementary Table S11 Associations between CHG-FI and risks of heart disease, stroke,

diabetes, and CMM using an alternative CKD definition (n = 6,812).

| **Exposure** | **Outcome** | **Crude model** | | **Model 1** | | **Model 2** | |
| --- | --- | --- | --- | --- | --- | --- | --- |
|  |  | **HR**  **(95% CI)** | ***P*-value** | **HR**  **(95% CI)** | ***P*-value** | **HR**  **(95% CI)** | ***P*-value** |
| CHG-FI | Heart disease | 1.46 (1.36~1.57) | <0.001 | 1.35 (1.25~1.47) | <0.001 | 1.35 (1.24~1.46) | <0.001 |
| CHG-FI tertile |  |  |  |  |  |  |  |
| T1 |  | 1(Ref) |  | 1(Ref) |  | 1(Ref) |  |
| T2 |  | 1.56 (1.34~1.81) | <0.001 | 1.55 (1.33~1.8) | <0.001 | 1.54 (1.32~1.79) | <0.001 |
| T3 |  | 2.19 (1.9~2.52) | <0.001 | 2.05 (1.76~2.38) | <0.001 | 2.02 (1.74~2.35) | <0.001 |
| P for trend |  |  | <0.001 |  | <0.001 |  | <0.001 |
| CHG-FI | Stroke | 1.93 (1.75~2.13) | <0.001 | 1.85 (1.66~2.07) | <0.001 | 1.85 (1.65~2.07) | <0.001 |
| CHG-FI tertile |  |  |  |  |  |  |  |
| T1 |  | 1(Ref) |  | 1(Ref) |  | 1(Ref) |  |
| T2 |  | 1.94 (1.52~2.47) | <0.001 | 1.88 (1.47~2.41) | <0.001 | 1.87 (1.46~2.39) | <0.001 |
| T3 |  | 2.95 (2.34~3.72) | <0.001 | 2.73 (2.14~3.48) | <0.001 | 2.69 (2.11~3.43) | <0.001 |
| P for trend |  |  | <0.001 |  | <0.001 |  | <0.001 |
| CHG-FI | Diabetes | 1.38 (1.26~1.5) | <0.001 | 1.31 (1.19~1.45) | <0.001 | 1.3 (1.18~1.43) | <0.001 |
| CHG-FI tertile |  |  |  |  |  |  |  |
| T1 |  | 1(Ref) |  | 1(Ref) |  | 1(Ref) |  |
| T2 |  | 1.4 (1.18~1.66) | <0.001 | 1.36 (1.14~1.62) | 0.001 | 1.34 (1.13~1.6) | 0.001 |
| T3 |  | 1.81 (1.53~2.13) | <0.001 | 1.69 (1.42~2.01) | <0.001 | 1.66 (1.39~1.97) | <0.001 |
| P for trend |  |  | <0.001 |  | <0.001 |  | <0.001 |
| CHG-FI | CMM | 1.89 (1.69~2.12) | <0.001 | 1.83 (1.62~2.07) | <0.001 | 1.81 (1.6~2.05) | <0.001 |
| CHG-FI tertile |  |  |  |  |  |  |  |
| T1 |  | 1(Ref) |  | 1(Ref) |  | 1(Ref) |  |
| T2 |  | 1.86 (1.41~2.44) | <0.001 | 1.84 (1.4~2.43) | <0.001 | 1.82 (1.38~2.4) | <0.001 |
| T3 |  | 3.28 (2.55~4.22) | <0.001 | 3.15 (2.41~4.11) | <0.001 | 3.05 (2.34~3.99) | <0.001 |
| P for trend |  |  | <0.001 |  | <0.001 |  | <0.001 |

Note: CKD was defined as self-report or estimated glomerular filtration rate (eGFR) < 60 mL/min/1.73 m², calculated using the CKD-EPI formula based on serum creatinine measurements, according to the KDIGO 2024 clinical practice guideline. Estimated glomerular filtration rate was estimated using the CKD-EPI formula:eGFR = 141 × min(SCr/k, 1)a × max(SCr /k, 1) - 1.209 × 0.993Age × 1.018 [if female] ×1.159 [if Black]: where k = 0.7 for females or 0.9 for males and a = -0.329 for females or -0.411 for males.

Data presented are HRs and 95% CIs.Model 1: adjusted for age, sex, marital status, educational level, residence, smoking status and drinking status.Model 2: adjusted for variables in Model 1 plus cancer,CKD,LDL-C, C-reactive protein,hemoglobin,and creatinine. Abbreviations:HR, hazard ratio; CI, confidence interval; Ref, reference;CKD: chronic kidney disease;LDL-C: low-density lipoprotein cholesterol;CHG-FI:cholesterol, high-density lipoprotein,  glucose and frailty indices; CMM:Cardiometabolic multimorbidity.

Supplementary Table S12 Excluding events occurring within the first two years of follow‑up

| **Exposure** | **Outcome** | **Crude model** | | **Model 1** | | **Model 2** | |
| --- | --- | --- | --- | --- | --- | --- | --- |
|  |  | **HR(95% CI)** | ***P*-value** | **HR(95% CI)** | ***P*-value** | **HR(95% CI)** | ***P*-value** |
| CHG-FI | Heart disease | 1.47 (1.36~1.58) | <0.001 | 1.36 (1.25~1.48) | <0.001 | 1.35 (1.24~1.47) | <0.001 |
| CHG-FI tertile | (n=6654) |  |  |  |  |  |  |
| T1 |  | 1(Ref) |  | 1(Ref) |  | 1(Ref) |  |
| T2 |  | 1.61 (1.37~1.89) | <0.001 | 1.6 (1.36~1.88) | <0.001 | 1.59 (1.35~1.87) | <0.001 |
| T3 |  | 2.22 (1.91~2.59) | <0.001 | 2.1 (1.79~2.47) | <0.001 | 2.07 (1.76~2.43) | <0.001 |
| P for trend |  |  | <0.001 |  | <0.001 |  | <0.001 |
| CHG-FI | Stroke | 1.94 (1.75~2.16) | <0.001 | 1.87 (1.67~2.1) | <0.001 | 1.87 (1.67~2.1) | <0.001 |
| CHG-FI tertile | (n=6781) |  |  |  |  |  |  |
| T1 |  | 1(Ref) |  | 1(Ref) |  | 1(Ref) |  |
| T2 |  | 1.96 (1.52~2.52) | <0.001 | 1.9 (1.47~2.46) | <0.001 | 1.89 (1.46~2.44) | <0.001 |
| T3 |  | 3.02 (2.38~3.84) | <0.001 | 2.81 (2.18~3.61) | <0.001 | 2.77 (2.15~3.56) | <0.001 |
| P for trend |  |  | <0.001 |  | <0.001 |  | <0.001 |
| CHG-FI | Diabetes | 1.35 (1.22~1.48) | <0.001 | 1.28 (1.15~1.42) | <0.001 | 1.26 (1.13~1.4) | <0.001 |
| CHG-FI tertile | (n=6743) |  |  |  |  |  |  |
| T1 |  | 1(Ref) |  | 1(Ref) |  | 1(Ref) |  |
| T2 |  | 1.39 (1.17~1.67) | <0.001 | 1.35 (1.13~1.61) | 0.001 | 1.34 (1.12~1.6) | 0.002 |
| T3 |  | 1.8 (1.52~2.13) | <0.001 | 1.68 (1.4~2.01) | <0.001 | 1.64 (1.37~1.96) | <0.001 |
| P for trend |  |  | <0.001 |  | <0.001 |  | <0.001 |
| CHG-FI | CMM | 1.89 (1.68~2.11) | <0.001 | 1.81 (1.6~2.05) | <0.001 | 1.78 (1.57~2.03) | <0.001 |
| CHG-FI tertile | (n=6800) |  |  |  |  |  |  |
| T1 |  | 1(Ref) |  | 1(Ref) |  | 1(Ref) |  |
| T2 |  | 1.88 (1.43~2.48) | <0.001 | 1.86 (1.4~2.45) | <0.001 | 1.83 (1.39~2.42) | <0.001 |
| T3 |  | 3.25 (2.51~4.2) | <0.001 | 3.1 (2.37~4.06) | <0.001 | 2.99 (2.28~3.92) | <0.001 |
| P for trend |  |  | <0.001 |  | <0.001 |  | <0.001 |

Data presented are HRs and 95% CIs.Model 1: adjusted for age, sex, marital status, educational level, residence, smoking status and drinking status.Model 2: adjusted for variables in Model 1 plus cancer,CKD,LDL-C, C-reactive protein,hemoglobin,and creatinine. Abbreviations:HR, hazard ratio; CI, confidence interval; Ref, reference;CKD: chronic kidney disease;LDL-C: low-density lipoprotein cholesterol;CHG-FI:cholesterol, high-density lipoprotein,  glucose and frailty indices; CMM:Cardiometabolic multimorbidity.

Supplementary Table S13 Competing-risk modeling was carried out using the Fine-Gray subdistribution hazards framework

| **Exposure** | **Total** | **n.event（%）** | **Crude model** | | **Adjust Model** | |
| --- | --- | --- | --- | --- | --- | --- |
|  |  |  | **HR(95% CI)** | ***P*-value** | **HR(95% CI)** | ***P*-value** |
| **Heart disease** |  |  |  |  |  |  |
| CHG-FI | 6812 | 1304 (19.1) | 1.44 (1.34~1.54) | <0.001 | 1.37 (1.27~1.48) | <0.001 |
| CHG-FI tertile |  |  |  |  |  |  |
| T1 | 2271 | 266 (11.7) | 1(Ref) |  | 1(Ref) |  |
| T2 | 2270 | 422 (18.6) | 1.58 (1.37~1.83) | <0.001 | 1.59 (1.37~1.84) | <0.001 |
| T3 | 2271 | 616 (27.1) | 2.18 (1.9~2.5) | <0.001 | 2.11 (1.83~2.44) | <0.001 |
| P for trend |  |  |  | <0.001 |  | <0.001 |
| **Stroke** |  |  |  |  |  |  |
| CHG-FI | 6812 | 554 (8.1) | 1.9 (1.72~2.09) | <0.001 | 1.85 (1.64~2.08) | <0.001 |
| CHG-FI tertile |  |  |  |  |  |  |
| T1 | 2271 | 100 (4.4) | 1(Ref) |  | 1(Ref) |  |
| T2 | 2270 | 180 (7.9) | 1.81 (1.42~2.3) | <0.001 | 1.77 (1.38~2.27) | <0.001 |
| T3 | 2271 | 274 (12.1) | 2.75 (2.19~3.45) | <0.001 | 2.62 (2.05~3.34) | <0.001 |
| P for trend |  |  |  | <0.001 |  | <0.001 |
| **T2DM** |  |  |  |  |  |  |
| CHG-FI | 6812 | 932 (13.7) | 1.32 (1.21~1.43) | <0.001 | 1.29 (1.17~1.42) | <0.001 |
| CHG-FI tertile |  |  |  |  |  |  |
| T1 | 2271 | 218 (9.6) | 1(Ref) |  | 1(Ref) |  |
| T2 | 2270 | 309 (13.6) | 1.41 (1.19~1.67) | <0.001 | 1.39 (1.17~1.65) | <0.001 |
| T3 | 2271 | 405 (17.8) | 1.74 (1.49~2.04) | <0.001 | 1.69 (1.43~2) | <0.001 |
| P for trend |  |  |  | <0.001 |  | <0.001 |
| **CMM** |  |  |  |  |  |  |
| CHG-FI | 6812 | 467 (6.9) | 1.89 (1.7~2.1) | <0.001 | 1.87 (1.65~2.13) | <0.001 |
| CHG-FI tertile |  |  |  |  |  |  |
| T1 | 2271 | 73 (3.2) | 1(Ref) |  | 1(Ref) |  |
| T2 | 2270 | 144 (6.3) | 2 (1.51~2.64) | <0.001 | 2 (1.5~2.66) | <0.001 |
| T3 | 2271 | 250 (11) | 3.42 (2.64~4.43) | <0.001 | 3.37 (2.56~4.43) | <0.001 |
| P for trend |  |  |  | <0.001 |  | <0.001 |

Data presented are HRs and 95% CIs.Model 1: adjusted for age, sex, marital status, educational level, residence, smoking status and drinking status.Model 2: adjusted for variables in Model 1 plus cancer,CKD,LDL-C, C-reactive protein,hemoglobin,and creatinine. Abbreviations:HR, hazard ratio; CI, confidence interval; Ref, reference;CKD: chronic kidney disease;LDL-C: low-density lipoprotein cholesterol;CHG-FI:cholesterol, high-density lipoprotein,  glucose and frailty indices; CMM:Cardiometabolic multimorbidity.

Death information was obtained from two sources: (1) for the 2011–2018 period, death data were extracted from the Harmonized CHARLS dataset using the RwIWSTAT variable; (2) for the 2019–2020 period, deaths were identified based on the year of death reported in the 2020 exit questionnaire. This approach follows the standard methodology established in prior CHARLS-based research for handling competing risks. Nonetheless, these death data may be subject to underreporting or incomplete ascertainment, which could bias mortality estimates (for example, through differential misclassification or censoring). Such limitations should be acknowledged when interpreting results.

Supplementary Table S14 E-values for the association between CHG-FI and heart disease, stroke, diabetes, and CMM

| **Outcome** | **Comparison** | **Adjusted HR (95% CI)** | **E-value (Point Estimate)** |
| --- | --- | --- | --- |
| Heart disease | T3 vs T1 | 2 (1.72~2.32) | 3.41 |
| Stroke | T3 vs T1 | 2.68 (2.10~3.42) | 4.80 |
| Diabetes | T3 vs T1 | 1.65 (1.38~1.96) | 2.69 |
| CMM | T3 vs T1 | 3.02 (2.31~3.95) | 5.49 |

Data presented are HRs and 95% CIs.Adjusted Model : adjusted for age, sex, marital status, educational level, residence, smoking status and drinking status,cancer,CKD,LDL-C, C-reactive protein, hemoglobin,and creatinine.

Abbreviations:HR, hazard ratio; CI, confidence interval; Ref, reference;CKD: chronic kidney disease;LDL-C: low-density lipoprotein cholesterol；CHG-FI:cholesterol, high-density lipoprotein,  glucose and frailty indices; CMM:Cardiometabolic multimorbidity.

Supplementary Table S15 Receiver operating characteristic (ROC) curve analysis for CHG‑FI and related indices in predicting heart disease, stroke, diabetes, and CMM

| **Variable** | **Outcome** | **AUC (95% CI ) (%)** | **Specificity** | **Sensitivity** |
| --- | --- | --- | --- | --- |
| CHG | Heart disease | 52.03(50.27 ~ 53.78) | 0.51 | 0.54 |
| FI |  | 60.40(58.73 ~ 62.07) | 0.49 | 0.68 |
| TyG-FI |  | 60.64(58.98 ~ 62.31) | 0.52 | 0.64 |
| CHG-FI |  | 60.64(58.97 ~ 62.31) | 0.48 | 0.69 |
| CHG | Stroke | 57.98(55.55 ~ 60.40) | 0.48 | 0.66 |
| FI |  | 62.31(59.91~ 64.70) | 0.42 | 0.75 |
| TyG-FI |  | 62.75(60.36 ~ 65.13) | 0.40 | 0.78 |
| CHG-FI |  | 63.02(60.64 ~ 65.40) | 0.40 | 0.78 |
| CHG | Diabetes | 61.91(59.96 ~ 63.86) | 0.61 | 0.57 |
| FI |  | 56.36(54.40 ~ 58.32) | 0.32 | 0.78 |
| TyG-FI |  | 57.22(55.26 ~ 59.17) | 0.40 | 0.71 |
| CHG-FI |  | 57.47(55.52 ~ 59.42) | 0.38 | 0.74 |
| CHG | CMM | 61.24(58.56 ~ 63.92) | 0.60 | 0.59 |
| FI |  | 62.81(60.25 ~ 65.37) | 0.41 | 0.77 |
| TyG-FI |  | 63.49(60.95 ~ 66.03) | 0.61 | 0.58 |
| CHG-FI |  | 63.80(61.27 ~ 66.34) | 0.67 | 0.54 |

AUC values are presented as percentages and 95% confidence intervals (CIs) are shown in parentheses. Reported sensitivity and specificity correspond to the optimal cutoff point (determined by maximizing the Youden index).

Abbreviations:CHG: Cholesterol, high‑density lipoprotein, glucose indices; FI: Frailty Index; TyG‑FI:Triglyceride‑glucose and frailty indices; CHG-FI:cholesterol, high-density lipoprotein,  glucose and frailty indices; CMM:Cardiometabolic multimorbidity.

Supplementary Table S16 Additional prognostic value of TyG-FI and CHG-FI for predicting CMM

| **Model** | **AUC(95% CI)** | ***P-*value** | **NRI (95% CI)** | ***P-*value** | **IDI (95% CI)** | ***P-*value** |
| --- | --- | --- | --- | --- | --- | --- |
| TG<150mg/dL | | | | | | |
| Basic model | 0.602 (0.570~0.634) |  | Ref |  | Ref |  |
| +TyG-FI | 0.649 (0.619~0.680) | <0.001 | 0.346(0.235~0.457) | <0.001 | 0.008(0.005~0.011) | <0.001 |
| +CHG-FI | 0.651 (0.620~0.682) | <0.001 | 0.364(0.253~0.475) | <0.001 | 0.008(0.005~0.012) | <0.001 |
| TG≥150mg/dL | | | | | | |
| Basic model | 0.646 (0.600~0.692) |  | Ref |  | Ref |  |
| +TyG-FI | 0.693 (0.650~0.736) | 0.019 | 0.284(0.109~0.458) | 0.001 | 0.019(0.007~0.030) | 0.001 |
| +CHG-FI | 0.694 (0.651~0.737) | 0.017 | 0.268(0.094~0.442) | 0.003 | 0.019(0.008~0.031) | 0.001 |

Basic model were adjusted for age, sex, marital status, educational level, residence, smoking status,drinking status,cancer,CKD,LDL-C, C-reactive protein, hemoglobin,and creatinine. Abbreviations:Ref, reference;CKD: chronic kidney disease;LDL-C: low-density lipoprotein cholesterol;TG:triglyceride;TyG‑FI:Triglyceride‑glucose and frailty indices; CHG-FI:cholesterol, high-density lipoprotein,  glucose and frailty indices; CMM:Cardiometabolic multimorbidity;AUC: Area Under the Curve;NRI:Net Reclassification Improvement; IDI: Integrated Discrimination Improvement.


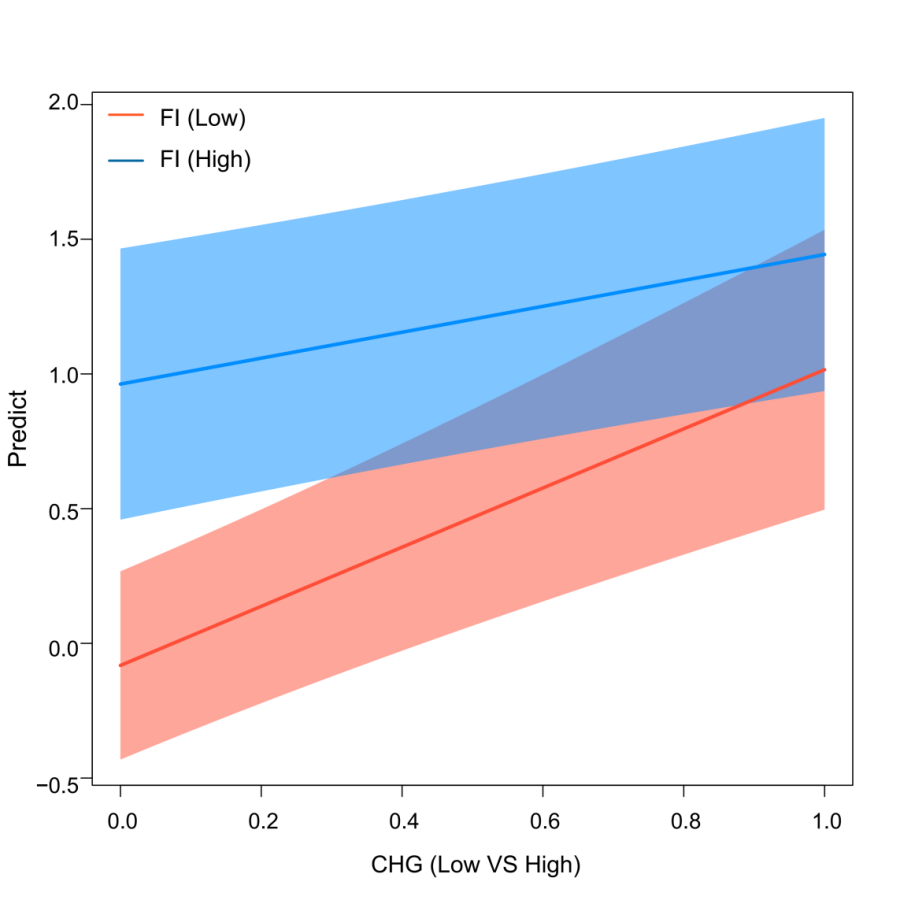


Supplementary Figure S1 Interaction between CHG and FI in the development of new-onset CMM Model was adjusted for age, sex, marital status, educational level, residence, smoking status,drinking status,cancer,CKD,LDL-C, C-reactive protein, hemoglobin,and creatinine.

Model-predicted risk of incident CMM across CHG (x-axis), by FI status (red: FI low; blue: FI high). Shaded bands = 95% CIs (multivariable-adjusted). Abbreviations:CHG: Cholesterol, high‑density lipoprotein, glucose indices; FI: Frailty Index;CKD: chronic kidney disease;LDL-C: low-density lipoprotein cholesterol.


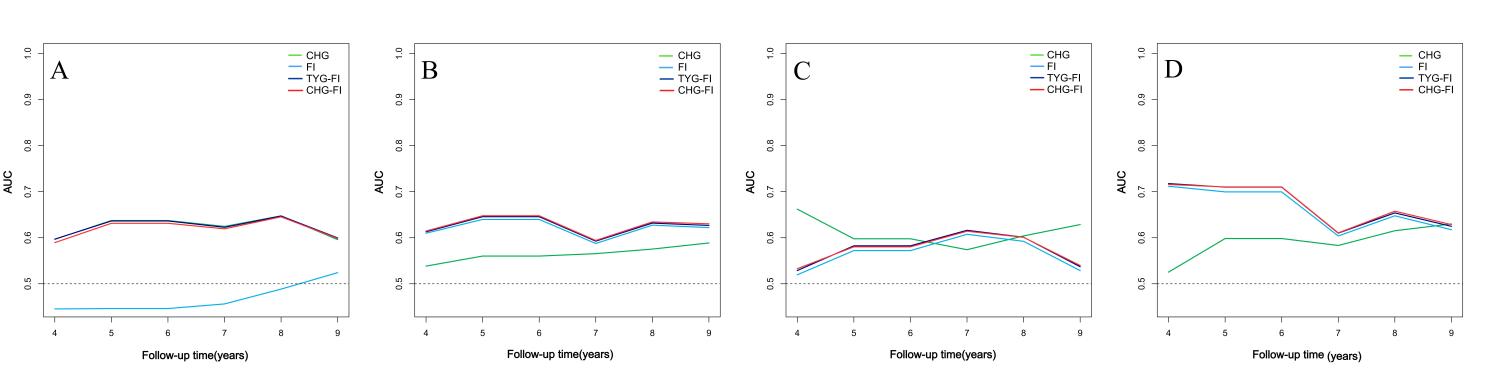


Supplementary Figure S2 Time‑dependent predictive performance of CHG‑FI and related measures for incident outcomes: heart disease (A), stroke (B), diabetes (C) and CMM (D) across follow‑up years 4–9 Curves denote CHG (green), FI (light blue), TyG‑FI (dark blue) and CHG‑FI (red); AUC :area under the time‑dependent ROC curve. The dashed horizontal line indicates AUC = 0.5 (no discrimination). AUCs were estimated at annual intervals using time‑to‑event ROC methodology that accounts for right censoring to depict temporal changes in discriminatory ability. Overall, combined indices (CHG‑FI and TyG‑FI) tended to show higher time‑dependent AUCs for CMM and heart disease than single measures.

Model was adjusted for age, sex, marital status, educational level, residence, smoking status,drinking status,cancer,CKD,LDL-C, C-reactive protein, hemoglobin,and creatinine.

Abbreviations:AUC, area under the curve; CMM, cardiometabolic multimorbidity;CHG:cholesterol,high-density lipoprotein, and glucose indices；FI:frailty index;TyG‑FI: Triglyceride‑glucose and frailty indices;CHG-FI:cholesterol, high-density lipoprotein,  glucose and frailty indices;CKD: chronic kidney disease;LDL-C: low-density lipoprotein cholesterol.
